# Chloride transporter KCC2-dependent neuroprotection depends on the N-terminal protein domain

**DOI:** 10.1038/cddis.2015.127

**Published:** 2015-06-04

**Authors:** A Winkelmann, M Semtner, J C Meier

**Affiliations:** 1 RNA Editing and Hyperexcitability Disorders Helmholtz Group, Max Delbrück Center for Molecular Medicine, Berlin 13125, Germany; 2 Division of Cell Physiology, TU Braunschweig, Zoological Institute, Braunschweig 38106, Germany

## Abstract

Neurodegeneration is a serious issue of neurodegenerative diseases including epilepsy. Downregulation of the chloride transporter KCC2 in the epileptic tissue may not only affect regulation of the polarity of GABAergic synaptic transmission but also neuronal survival. Here, we addressed the mechanisms of KCC2-dependent neuroprotection by assessing truncated and mutated KCC2 variants in different neurotoxicity models. The results identify a threonine- and tyrosine-phosphorylation-resistant KCC2 variant with increased chloride transport activity, but they also identify the KCC2 N-terminal domain (NTD) as the relevant minimal KCC2 protein domain that is sufficient for neuroprotection. As ectopic expression of the KCC2-NTD works independently of full-length KCC2-dependent regulation of Cl^−^ transport or structural KCC2 C-terminus-dependent regulation of synaptogenesis, our study may pave the way for a selective neuroprotective therapeutic strategy that will be applicable to a wide range of neurodegenerative diseases.

Neurodegeneration restricts neuron numbers during development but can become a serious issue in disease conditions such as temporal lobe epilepsy (TLE).^1^ GABA-activated Cl^−^ channels contribute to activity-dependent refinement of neural networks by triggering the so-called giant depolarizing potentials providing developing neurons with a sense of activity essential for neuronal survival and co-regulation of excitatory glutamatergic and (inhibitory) GABAergic synapses.^2^ By regulating transmembrane Cl^−^ gradients KCC2 plays a vital role in development and disease.^3^ In addition, KCC2 plays a protein structural role in spine formation through its C-terminal protein domain (CTD).^4, 5^ Hence, regulation of KCC2 expression and function is relevant for development and disease-specific plasticity of neural networks.^6, 7, 8, 9^

GlyR *α*3K RNA editing leads to proline-to-leucine substitution (P185L) in the ligand-binding domain and generates gain-of-function neurotransmitter receptors.^10, 11, 12, 13^ GlyR RNA editing is upregulated in the hippocampus of patients with TLE and leads to GlyR *α*3K^185L^-dependent tonic inhibition of neuronal excitability associated with neurodegeneration.^14^ KCC2 expression promotes neuroprotection^14, 15^ but whether this involves regulation of transmembrane Cl^−^ gradient or protein structural role is a matter of debate.^14, 15^

Here, we assessed neuroprotection through several KCC2 variants in two different models of neurodegeneration including chronic neuronal silencing (*α*3K^185L^ model) and acute neuronal overexcitation (NMDA model).^14, 15^ The results identify a threonine- and tyrosine-phosphorylation-resistant KCC2 variant with increased Cl^−^ transport activity, but they also demonstrate that the N-terminal KCC2 protein domain (NTD) is sufficient for neuroprotection.

## Results

**Figure 1:**
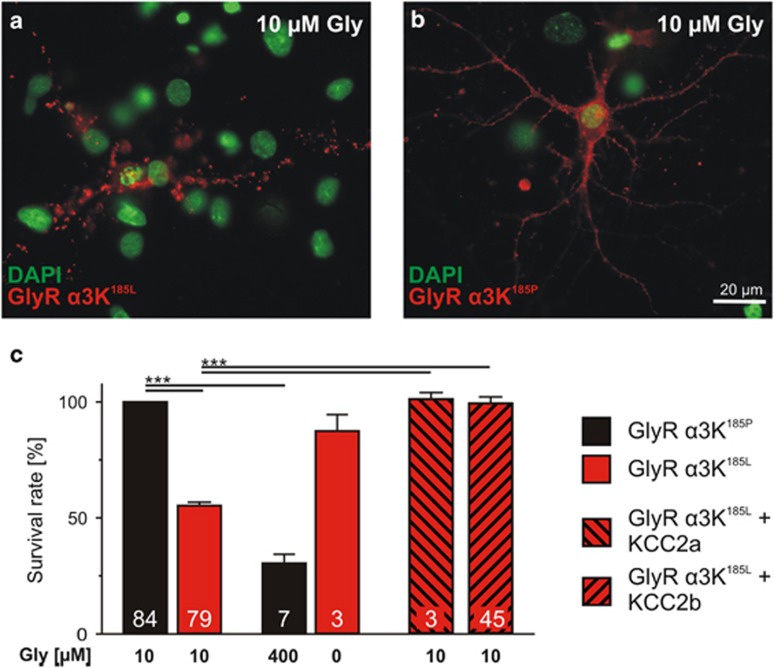
GlyR activation-dependent neurodegeneration and KCC2-dependent neuroprotection. Images of neurons with surface-stained GlyR *α*3K^185L^ (**a**) and GlyR *α*3K^185P^ (**b**). According to fragmented dendrites and pyknotic nuclei, neurodegeneration is obvious in neurons with continuous GlyR activation (**a**). (**c**) Quantification of the fraction of surviving neurons in each condition reveals that neurodegeneration occurs only under GlyR *α*3K-activating conditions and that co-expression of KCC2 (either splice variant KCC2a or KCC2b) protects neurons with continuous GlyR *α*3K^185L^ activation. Numbers in the bar graphs indicate the number of cultures analyzed. For KCC2 protein structure see Supplementary Figure 1. For values, see Supplementary Table 1. ***P <0.001

We first investigated the mechanisms underlying the neurotoxic effects of the RNA-edited GlyR *α*3K variant (*α*3K^185L^) on primary hippocampal neurons as described.^14^ Neurons were transfected at day *in vitro* (DIV) 6 either with non-edited (185P; control) or RNA-edited GlyR *α*3K (185L) and maintained for 3 days in 10 *μ*M glycine, a concentration that should selectively activate GlyR *α*3K^185L^.^10^ As shown previously,^14^ ~50% of the GlyR *α*3K^185L^-expressing neurons exhibited fragmented dendrites and pyknotic nuclei (Figures 1a and c; Supplementary Table 1), both clear indicators of neurodegeneration, whereas overexpression of non-edited GlyR *α*3K^185P^ in 10 *μ*M glycine had little effect on neuronal survival (Figures 1b and c). Appearance of fragmented dendrites was not due to rapid internalization of surface-labeled GlyR *α*3K^185L^ because comparable fluorescent signals were obtained upon cell permeabilization (not shown). To further investigate the possibility that GlyR activation is responsible for neurodegeneration, GlyR *α*3K^185P^-expressing neurons were maintained under GlyR-activating conditions (400 *μ*M glycine), and GlyR *α*3K^185L^-expressing neurons under non-activating (0 *μ*M glycine) conditions.^10^ In agreement with different apparent glycine affinities of non-edited GlyR *α*3K^185P^ and edited GlyR *α*3K^185L^^10^ the data in Figure 1c clearly show that glycine-dependent activation of GlyR *α*3K triggers neurodegeneration.

Expression of KCC2 is inversely correlated to GlyR RNA-editing in TLE hippocampi.^14^ Two KCC2 RNA splice variants with different N-termini are known^16^ (Supplementary Figure 1A), but neuroprotective effects were demonstrated for KCC2b only.^14, 15^ We therefore tested whether KCC2a and KCC2b protect neurons against GlyR *α*3K^185L^-mediated degeneration (Figure 1c). KCC2a- and KCC2b-positive neurons were identified with 2A self-processing peptide-coupled EGFP (Supplementary Figure 1B). Co-expression of KCC2a and KCC2b rescued the survival of neurons cultured under GlyR *α*3K^185L^-activating conditions (Figure 1c; Supplementary Table 1). This result confirms our previous data^14^ and furthermore reveals that KCC2-dependent neuroprotection does not depend on alternative splicing of exon 1.^16^ KCC2b was henceforth used and referred to as ‘KCC2wt'.

### Role of spontaneous neural network activity for GlyR-dependent neurodegeneration

**Figure 2:**
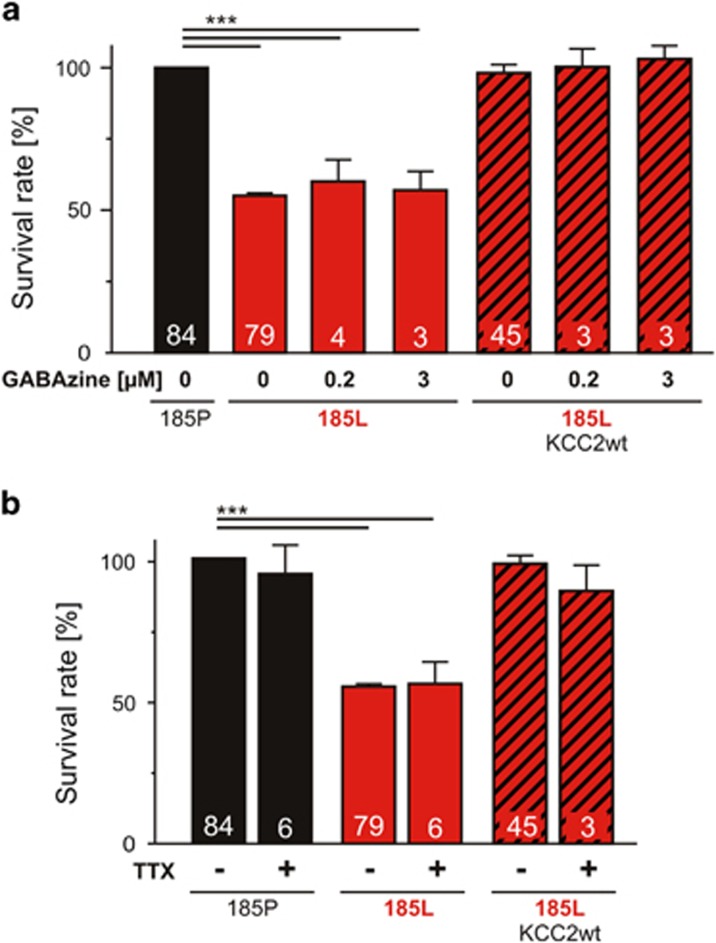
Spontaneous neuronal network activity does not play a role in GlyR-dependent neurodegeneration. Quantification of the effects of GABAzine (**a**) and TTX (1 *μ*M, **b**) on the survival of neurons with activated GlyR *α*3K^185L^. Two different GABAzine concentrations (0.2 and 3 *μ*M) were used to block synaptic or synaptic and non-synaptic GABA_A_R activation. Numbers in the bar graphs indicate the number of cultures analyzed. For values, see Supplementary Table 1. Statistical significance is indicated with ***P<0.001

Spontaneous neuronal activity at the beginning of experimental GlyR *α*3K^185L^ expression period is mainly dependent on GABA_A_R activation (Supplementary Figure 2), suggesting that inability of chronically silent neurons with enduring GlyR *α*3K^185L^ activation^14^ to participate in spontaneous neural network activity underlies neurodegeneration. To address this possibility, we blocked either synaptic or synaptic and non-synaptic GABA_A_Rs, as described,^17^ using 0.2 *μ*M or 3 *μ*M of the competitive GABA_A_R antagonist GABAzine. However, neither 0.2 *μ*M nor 3 *μ*M GABAzine rescued chronically silent neurons, and KCC2wt-dependent neuroprotection did not require GABA_A_R activation (Figure 2a; Supplementary Table 1). Furthermore, neurodegeneration/neuroprotection did not require action-potential-dependent synaptic transmission because tetrodotoxin (TTX) had no effect on survival (Figure 2b; Supplementary Table 1). These results suggest that intrinsic cellular mechanisms are responsible for neurodegeneration rather than inability of chronically silent neurons to participate in spontaneous neural network activity.

### Role of intracellular Ca^2+^ concentration for GlyR-dependent neurodegeneration

**Figure 3:**
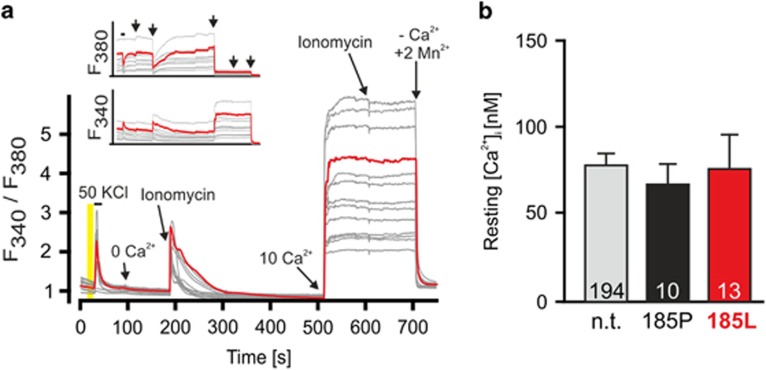
Continuous GlyR *α*3K^185L^ activation does not affect resting [Ca^2+^]_i_. Intracellular Ca^2+^ concentration at rest ([Ca^2+^]_i_) was determined according to the procedure described by Jung *et al.*^20^ (**a**) Neurons were loaded 2–3 days after transfection with fura-2. Red and gray traces correspond to signals obtained from a GlyR *α*3K^185L^-positive neuron (red) and non-transfected neurons (gray) in the neighborhood of the transfected neuron in the same viewfield. A solution with 50 mM KCl was applied to monitor the viability of the cells according to their response with regard to fura-2 signals. Cells that did not respond to 50 mM KCl with changes in the F_340_/F_380_ ratio were not included in the determination of resting [Ca^2+^]_i_. Resting [Ca^2+^]_i_ was determined within the 10 s (marked with a yellow bar) prior to the application of 50 mM KCl. To obtain minimal (0 mM) and maximal (10 mM) Ca^2+^ signals for calibration, cells were permeabilized with either 10 *μ*M ionomycin or 10 *μ*M 4-bromo-antibiotic A23187. 2 mM Mn^2+^ were applied to quench the signal at the end of each experiment and to obtain background fluorescence that was subtracted from all F340 and F380 values. (**b**) GlyR *α*3K^185L^ expression and activation in the presence of 10 *μ*M glycine does not affect resting [Ca^2+^]_i_. Numbers in the bar graphs indicate the number of neurons analyzed

Activation of non-synaptic NMDA receptors (NMDARs) and Ca^2+^-activated Cl^−^ channel Clca1 were implicated in neurodegeneration,^18^ and changes in resting intracellular Ca^2+^ concentration ([Ca^2+^]_i_) are involved in excitotoxicity.^19^ Thus, GlyR *α*3K^185L^-dependent neuronal intrinsic mechanisms of neurodegeneration may involve changes in intracellular Ca^2+^ homeostasis. To investigate this possibility, [Ca^2+^]_i_ of neurons expressing GlyR *α*3K^185L^ or GlyR *α*3K^185P^ in 10 *μ*M glycine (i.e., receptor-activating and non-activating conditions) were determined using fura-2, as described by Jung *et al.*^20^ (Figure 3a). However, [Ca^2+^]_i_ was comparable in all conditions as [Ca^2+^]_i_ in neurons with activated GlyR *α*3K^185L^ (78.9±19.2 nM, *n*=13) were similar to those in neurons with non-activated GlyR *α*3K^185P^ (69.4±11.1 nM, *n*=10; *P*=0.7125) or untransfected neurons (80.6±6.2 nM, *n*=194, *P*=0.9280; Figure 3b). These results rule out increased [Ca^2+^]_i_ as a cause of GlyR *α*3K^185L^ activation-dependent neurodegeneration.

### Enduring GlyR *α*3K^185L^ activation persistently changes neuronal intrinsic membrane properties

We next investigated neuronal intrinsic membrane properties including membrane resistance (R_N_) and the reversal potential of all membrane conductances, henceforth termed ‘membrane reversal potential' (V_rev_), a value of transmembrane voltage at which ionic diffusive and electrical forces counterbalance and no net transmembrane current is observed. We used gramicidin-perforated patch clamp because under this condition the intracellular milieu is not dependent on the recording pipette solution but on ion channels open at a given time of recording. We used voltage-clamp configuration and voltage pulses (−5 mV) starting from the holding potential (−50 mV) to determine R_N_. Voltage ramps (from −100 to −30 mV, 0.5 mV/ms; 140 ms total duration) were used to determine V_rev_ as exactly as possible according to reversal of current–voltage (IV) relationships. To determine V_rev_ of GABA_A_R-dependent currents (E_GABA_) IV curves measured in the presence and absence of GABA were subtracted. The fluorescent dye lucifer yellow and high Cl^−^ concentration in the pipette solution allowed monitoring stability of the perforated patch configuration (Supplementary Figure 3A). Neurons with GlyR *α*3K^185L^ expression were identified according to co-expression of mCherry fluorescent protein (Supplementary Figure 3A).

**Figure 4:**
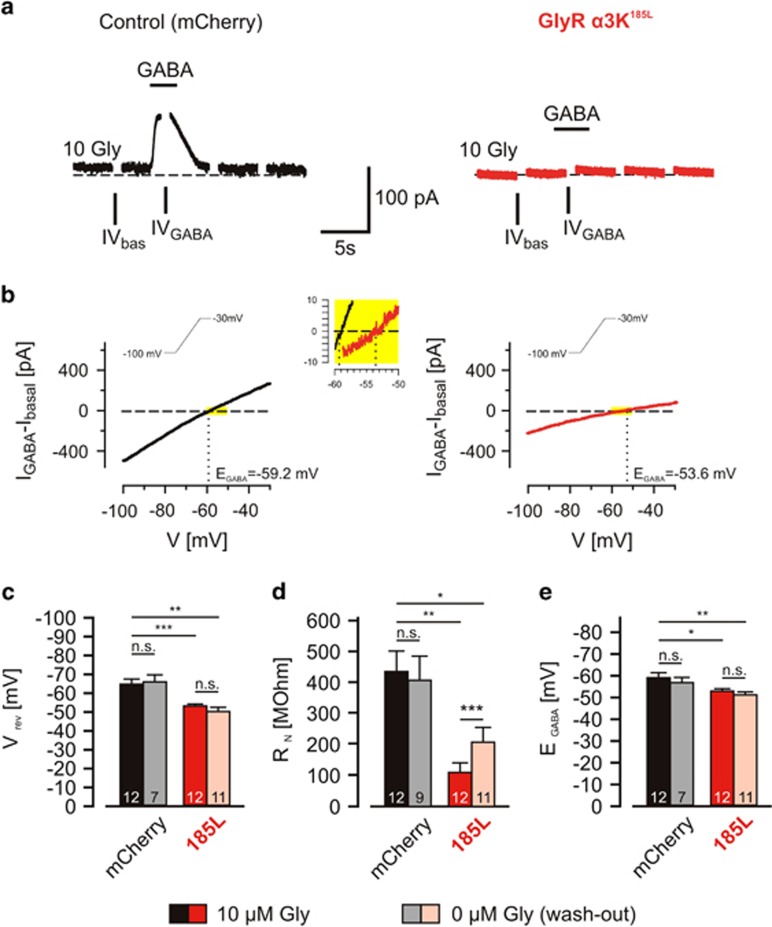
Intrinsic membrane properties of neurons with enduring GlyR *α*3K^185L^ activation. (**a**) Primary hippocampal neurons were either transfected with mCherry alone or with mCherry and GlyR *α*3K^185L^ (185L) and cultured for 2–3 days under receptor-activating conditions (10 *μ*M glycine). Neurons were investigated by perforated patch clamp (gramicidin) and voltage clamped at −50 mV. Under these conditions, the application of 100 *μ*M GABA lead to hyperpolarizing currents in most of the control neurons (**a**, left). GABA-evoked currents at the holding potential of −50 mV in GlyR *α*3K^185L^-expressing neurons were strongly diminished (**a**, right). Current–voltage (IV) relationships were obtained by applying voltage ramps from −100 to −30 mV every 5 s. Note that current traces during the voltage ramps are not shown. The time points for determination of IV_GABA_ and IV_bas_ are indicated. (**b**) The IVs of GABA-evoked currents were determined by subtracting the IVs in the presence and absence of GABA. Traces from individual cells are shown. The yellow-boxed high-power view between the two IV plots shows magnified IV curves of the depicted areas from recordings of the control cell left hand (black trace) and the GlyR *α*3K^185L^-positive cell right hand (red trace) in the presence of 10 *μ*M glycine. (**c**–**e**) The perforated patch-clamp experiments revealed that R_N_, V_rev_ and E_GABA_ were decreased in neurons with continuous GlyR *α*3K^185L^ activation. Note that washout of glycine from the extracellular solution only partially rescued R_N_, V_rev_ and E_GABA_. Numbers in the bar graphs indicate the number of neurons analyzed. Statistical significance is indicated with *P<0.05, **P<0.01 and ***P<0.001

We first analyzed IV relationships in the absence of GABA, that is, under basal conditions (Figure 4a, ‘IV_bas_'), in control neurons (mCherry only) and in neurons with activated GlyR *α*3K^185L^ and determined V_rev_. In neurons with enduring GlyR *α*3K^185L^ activation, V_rev_ was significantly more depolarized than in control neurons (Figures 4a and c; −52.1±1.1 mV, *n*=12 *versus* −63.6±2.7 mV, *n*=12; *P*=0.0005). Furthermore, R_N_ of neurons with enduring GlyR *α*3K^185L^ activation was significantly decreased compared to control neurons (Figure 4d; 108±31 MΩ, *n*=12 *versus* 429±65 MΩ, *n*=12; *P*=0.0012), indicating presence of open GlyR *α*3K^185L^ channels in the plasma membranes of neurons exposed to 10 *μ*M glycine.

**Figure 5:**
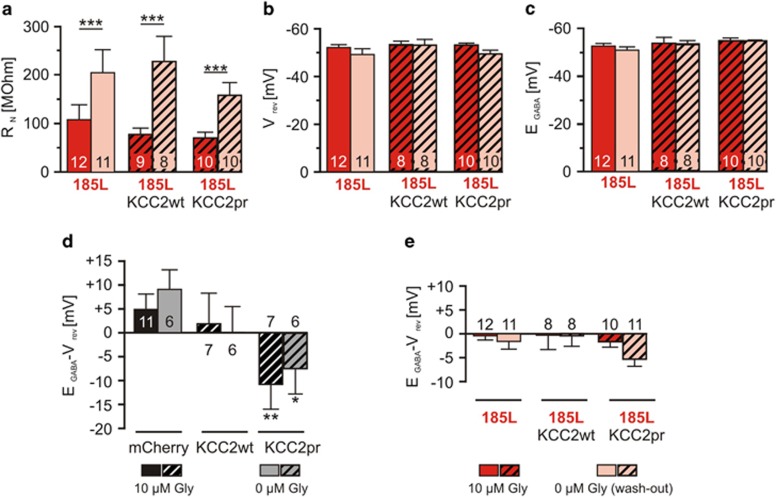
KCC2 does not rescue neuronal intrinsic membrane properties of neurons with enduring GlyR *α*3K^185L^ activation. Primary hippocampal neurons were either co-transfected with GlyR *α*3K^185L^ and mCherry, KCC2wt-2A-mCherry or KCC2pr-2A-mCherry (Supplementary Figure 1B) and cultured for 2–3 days in the presence of 10 *μ*M glycine (Gly). Hatched bars correspond to conditions with KCC2 co-expression, and for the purpose of direct comparison red and faded red bars shown in Figures 4c–e are shown here again. R_N_ (**a**), V_rev_ (**b**) and E_GABA_ (**c**) were determined with gramicidin-perforated patch-clamp experiments in the voltage-clamp mode. Note that co-expression of KCC2wt or KCC2pr had no significant effect on any of the investigated parameters that reflect neuronal intrinsic membrane properties. (**d** and **e**) Calculated driving forces for GABA_A_R responses. (**d**) Summary of the driving forces for GABA_A_R responses of cultured hippocampal neurons transfected with either mCherry, KCC2wt-2A-mCherry or KCC2pr-2A-mCherry, calculated by subtraction of V_rev_ from EGABA values. See Supplementary Figure 1 for constructs and Supplementary Figure 4 for sample traces from KCC2-2A-mCherry-expressing neurons. Compared to mCherry-transfected neurons, KCC2wt expression had no significant effect on the GABA driving forces of DIV 8–10 hippocampal neurons. In contrast, overexpression of KCC2pr significantly shifted the driving forces for GABA_A_R responses toward more negative values. The change in the driving forces is due to a negative shift of E_GABA_ (Supplementary Figure 4), not of V_rev_, indicating that the intracellular Cl^−^ concentration was changed by KCC2pr, not by KCC2wt, after 2–4 days of overexpression. Note that the calculated GABA_A_R driving forces were not dependent on the extracellular glycine concentration (0 or 10 *μ*M) during the perforated patch-clamp experiments. (**e**) Same as **d**, except that GlyR *α*3K^185L^ was co-expressed with mCherry, KCC2wt-2A-mCherry or KCC2pr-2A-mCherry. Owing to the large GlyR *α*3K^185L^-dependent Cl^−^ shunt all calculated driving forces were close to 0 mV. Numbers in the bar graphs indicate the number of neurons analyzed. Statistical significance is indicated with *P<0.05, **P<0.01 and ***P<0.001

We next determined E_GABA_ by subtracting IV curves measured in the presence (IV_GABA_) and absence (IV_bas_) of 100 *μ*M GABA (Figures 4a, b and e^21^). E_GABA_ values should be a measure of V_rev_ of Cl^−^ currents because GABA_A_Rs are like GlyRs permeable for Cl^−^. GABA_A_Rs are also permeable for HCO_3_^−^ (P_HCO3_/P_Cl_∼0.5–0.6^22, 23^), but as our solutions were not based on CO_2_/HCO_3_^−^, the determined E_GABA_ values primarily reflect V_rev_ of Cl^−^, that is E_Cl_. Most of the control neurons displayed hyperpolarizing currents at −50 mV in 100 *μ*M GABA (Figure 4a, left hand), which reversed at −58.7±2.3 mV (*n*=12; Figures 4b). In contrast, in neurons with enduring GlyR *α*3K^185L^ activation, GABA responses at the holding potential (−50 mV) were shunted (Figure 4a right hand; Supplementary Figure 3B right hand), and IV_GABA_ reversed close to that holding potential (−52.5 ± 1.1 mV, *n*=12, *P*=0.0195 compared to control, Figure 4b right hand, Figure 4e). Consequently, the calculated driving force of GABA responses was 4.9±2.9 mV in control neurons (*n*=12; Figure 5d), whereas it was close to 0 mV (−0.4±0.9 mV, *n*=12; Figure 5e) in neurons with enduring GlyR *α*3K^185L^ activation. Together with the strongly decreased R_N_ (Figure 4d), we conclude that enduring GlyR *α*3K^185L^ activation leads to large permanent Cl^−^ conductance that shunts GABA-dependent currents due to adaptation of transmembrane Cl^−^ gradient according to imposed membrane potential.

We next asked whether acute glycine washout during recording of neurons with activated GlyR *α*3K^185L^ would have effects on V_rev_ and E_GABA_ (Figures 4c–e). However, V_rev_ and E_GABA_ remained at more depolarized potentials compared to control neurons (−49.2±2.4 mV for V_rev_, −50.8±1.5 mV for E_GABA_, *n*=11), and R_N_ recovered only partially (Figure 4d; 107.8±30.5 MΩ, *n*=12 in 10 *μ*M glycine *versus* 203.9±47.2 MΩ, *n*=11 after glycine washout; *P*=0.0006). As GABA_A_R-dependent currents were observed upon glycine washout (Supplementary Figure 3B, right hand) GABA_A_R downregulation as a reason for observed effects regarding E_GABA_ can be ruled out. Thus, enduring GlyR *α*3K^185L^ activation during 3 days induced long-lasting changes in neuronal intrinsic membrane properties, which might involve changes in permeability for different ions including, besides Cl^−^, also K^+^, a well known major driving force of resting membrane potential generation.

### Enduring GlyR *α*3K^185L^ activation persistently changes resting membrane potential

We finally investigated resting membrane potential (V_m_) in current-clamp configuration (Supplementary Figures 3C and D). We clamped current at 0 pA and determined apparent V_m_ of neurons with continuous GlyR *α*3K^185L^ activation in 10 *μ*M glycine. Actually, V_m_ of GlyR *α*3K^185L^-expressing neurons in 10 *μ*M glycine required 2–3 min to stabilize after switching from voltage- to current-clamp configuration, and V_m_ slowly depolarized under these conditions from values around −50 mV (i.e., the formerly imposed holding potential) to values determined in current-clamp configuration reflecting apparent V_m_ (Supplementary Figure 3C). These slow adaptations of membrane potential consistently occurred only in 10 *μ*M glycine and in GlyR *α*3K^185L^-expressing neurons indicating that outwardly directed Cl^−^ currents through GlyR *α*3K^185L^ slowly decrease [Cl^−^]_i_ and thereby depolarize V_m_. Hence, these experiments revealed that V_m_ was significantly more depolarized in neurons with activated GlyR *α*3K^185L^ compared to control neurons (Supplementary Figure 3D; −35.9±2.6 mV, *n*=5 *versus* −51.2±2.4 mV, *n*=9; *P*=0.0104). As acute glycine washout significantly influenced V_m_ (Supplementary Figure 3D; −35.9±2.6 mV, *n*=5 in 10 *μ*M glycine *versus* −44.8±2.2 mV, *n*=10 upon glycine washout; *P*=0.0026), while no differences were observed in control neurons (Supplementary Figure 3D; −52.6±2.4 mV, *n*=8 in 10 *μ*M glycine *versus* −51.2±2.4 mV, *n*=7 upon glycine washout; *P*=0.6953), these data suggest that enduring GlyR *α*3K^185L^ activation shifts V_m_ toward E_Cl_. After glycine washout, V_m_ remained more depolarized in GlyR *α*3K^185L^-expressing neurons compared to control neurons (*P*=0.0306), and hence as discussed above, these results consolidate conclusion that enduring GlyR *α*3K^185L^ activation induced long-lasting changes of neuronal intrinsic membrane properties. A priori, these results suggest that KCC2 expression may prevent persistent changes in intrinsic membrane properties and possibly involve Cl^−^ transport in neuroprotection, as suggested earlier.^14, 15^

### KCC2 Cl^−^ transport activity is not relevant for neuroprotection

**Figure 6:**
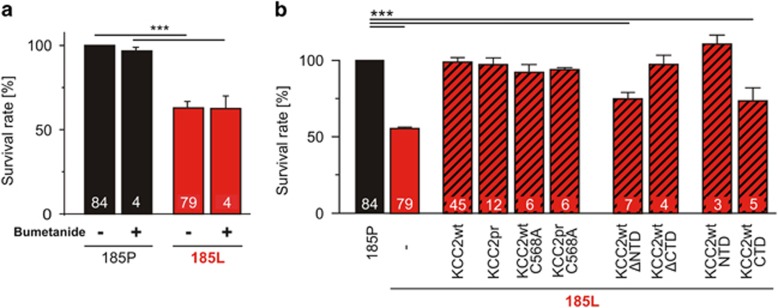
Protein structural role of KCC2 in neuroprotection. (**a**) The antagonist bumetanide (10 *μ*M) of the Cl^−^ transporter NKCC1 has no effect on neuronal survival irrespectively of whether hippocampal neurons were challenged with tonic GlyR *α*3K^185L^ activation or not. (**b**) The Cl^−^ transport-deficient KCC2wt/pr-C568A as well as the C-terminally truncated KCC2-ΔCTD were neuroprotective, whereas KCC2 constructs lacking the N-terminal domain (NTD) or containing only the isolated KCC2-CTD were not neuroprotective. Note that the isolated KCC2-NTD was sufficient to mediate full rescue of neuronal survival. Numbers in the bar graphs indicate the number of cultures analyzed. For values, see Supplementary Table 1. Statistical significance is indicated with ***P<0.001

Gramicidin-perforated patch clamp was used again to analyze the effects of co-expression of KCC2wt on membrane properties of neurons with enduring GlyR *α*3K^185L^ activation. KCC2-positive neurons were identified according to 2A-self-cleaving peptide-coupled mCherry (Supplementary Figure 1B). KCC2wt was not able to substantially change neuronal intrinsic membrane properties of GlyR *α*3K^185L^-expressing neurons (Figure 5). Similar to mCherry/GlyR *α*3K^185L^-expressing neurons in 10 *μ*M glycine (Figure 4), R_N_ remained significantly decreased (77.6±12.8 MΩ, *n*=9, Figure 5a), and V_rev_ and E_GABA_ also shifted toward imposed holding potential of −50 mV when KCC2wt was co-expressed (−53.4±1.4 mV and −53.7±2.5 mV, respectively, *n*=8, Figures 5b and c; calculated driving forces for GABA_A_R responses, Figure 5e). This suggests that KCC2wt Cl^−^ transport activity was not sufficient to overcome GlyR *α*3K^185L^-mediated Cl^−^ conductance. Moreover, upon acute glycine washout, V_rev_ and E_GABA_ remained largely unchanged (Figures 5b and c), and R_N_ recovered only partially (Figure 5a), similar to neurons with enduring GlyR *α*3K^185L^ activation in the absence of KCC2wt (Figure 4d). Thus, co-expression of KCC2wt was not able to prevent persistent changes in intrinsic membrane properties of neurons with enduring GlyR *α*3K^185L^ activation. For control purpose, we checked KCC2 Cl^−^ transport functionality and recorded KCC2wt-positive neurons in the absence of GlyR *α*3K^185L^ expression (Supplementary Figure 4). Surprisingly, no significant differences in E_GABA_ were found between control neurons (E_GABA_: −58.7±2.3 mV, *n*=12) and those expressing KCC2wt (E_GABA_: −55.4±2.8 mV, *n*=7, *P*=0.3920; Supplementary Figure 4B and Figure 5d). However, a mutant variant of KCC2 (‘KCC2pr' Supplementary Figure 1A), which should be resistant to phosphorylation-dependent downregulation of Cl^−^ transport activity^24, 25^ shifted E_GABA_ in neurons without GlyR *α*3K^185L^ co-expression (KCC2pr: −65.8±1.5 mV, *n*=8; *P*=0.0325 *versus* control: −58.7±2.3 mV, *n*=12; *P*=0.0049 *versus* KCC2wt: −55.4±2.8 mV, *n*=7; Supplementary Figures 4A and B; Figure 5d). These results were confirmed using recording of intracellular Ca^2+^ dynamics with Oregon Green 488 (Life Technologies, Darmstadt, Germany) in response to GABA application in more immature hippocampal neurons at DIV 6–7 (Supplementary Figure 5). Again, KCC2wt was not able to prevent GABA-induced increases in intracellular Ca^2+^ signals in hippocampal neurons without GlyR *α*3K^185L^ co-expression (F_GABA_/F_KCl_: 0.21±0.04, *n*=29 for KCC2wt *versus* 0.23±0.02, *n*=114 for untransfected neurons, *P*=0.7666; Supplementary Figure 5C), whereas the KCC2pr mutant was effective (F_GABA_/F_KCl_: 0.12±0.03, *n*=29 for KCC2pr *versus* 0.22±0.02, *n*=110 for untransfected neurons, *P*=0.0107; Supplementary Figure 5C). Importantly, KCC2wt and KCC2pr Cl^−^ transport activities were apparent in primary cortical neurons under these conditions (F_GABA_/F_KCl_: 0.11±0.03, *n*=36 for KCC2wt *versus* 0.40±0.03, *n*=158 for untransfected neurons, *P*<0.001; F_GABA_/F_KCl_: 0.08±0.03, *n*=44 for KCC2pr *versus* 0.50±0.03, *n*=188 for untransfected neurons, *P*<0.001; Supplementary Figure 5D). Hence, KCC2wt is a functional Cl^−^ transporter, and cortical and hippocampal neurons reveal phosphorylation-dependent differences in the regulation of KCC2 Cl^−^ transport activity. However, because KCC2wt protected hippocampal neurons against GlyR *α*3K^185L^ activation-dependent neurodegeneration (Figure 1), these results also suggest that Cl^−^ extrusion is not relevant to KCC2-dependent neuroprotection. Indeed, block of Cl^−^ import activity through NKCC1 using bumetanide (10 *μ*M) also failed to rescue neuronal survival (Figure 6a).

### The KCC2-NTD mediates neuroprotection in the GlyR *α*3K^185L^ model of neurodegeneration

KCC2 was previously shown to have a protein structural role for synaptogenesis by interaction with cytoskeleton-associated protein 4.1N.^5, 26^ This finding encouraged us to clarify if cytoskeletal signaling is involved in neuroprotection, but KCC2-C568A mutant which is unable to interact with protein 4.1N and Cl^−^ transport-deficient^5, 26^ rescued survival of neurons with continuous GlyR *α*3K^185L^ activation as well as KCC2wt (Figure 6b; Supplementary Table 1). Furthermore, KCC2pr-C568A rescued neuronal survival indicating that KCC2-dependent neuroprotection is independent of phosphorylation of Y903, T906, T1007 and Y1087 in the KCC2-CTD (Supplementary Figure 1A). These results reveal that KCC2-dependent neuroprotection is independent of protein 4.1N-dependent cytoskeletal signaling and further strengthen our conclusion that KCC2-dependent neuroprotection does not rely on Cl^−^ transport. They also suggest that it is not the KCC2-CTD, which mediates neuroprotection. Indeed, deletion of NTD, not of CTD, abolished KCC2-dependent neuroprotection (Figure 6b; Supplementary Table 1). Reciprocally, co-expression of the KCC2-NTD, not of KCC2-CTD, rescued survival of neurons with enduring GlyR *α*3K^185L^ activation (Figure 6b). That KCC2-ΔNTD or KCC2-CTD failed to protect neurons was not due to poor protein expression (Supplementary Figure 6). These results identify KCC2-NTD as relevant neuroprotective signaling domain.

### KCC2-NTD mediates neuroprotection in the NMDA-dependent model of excitotoxicity

**Figure 7:**
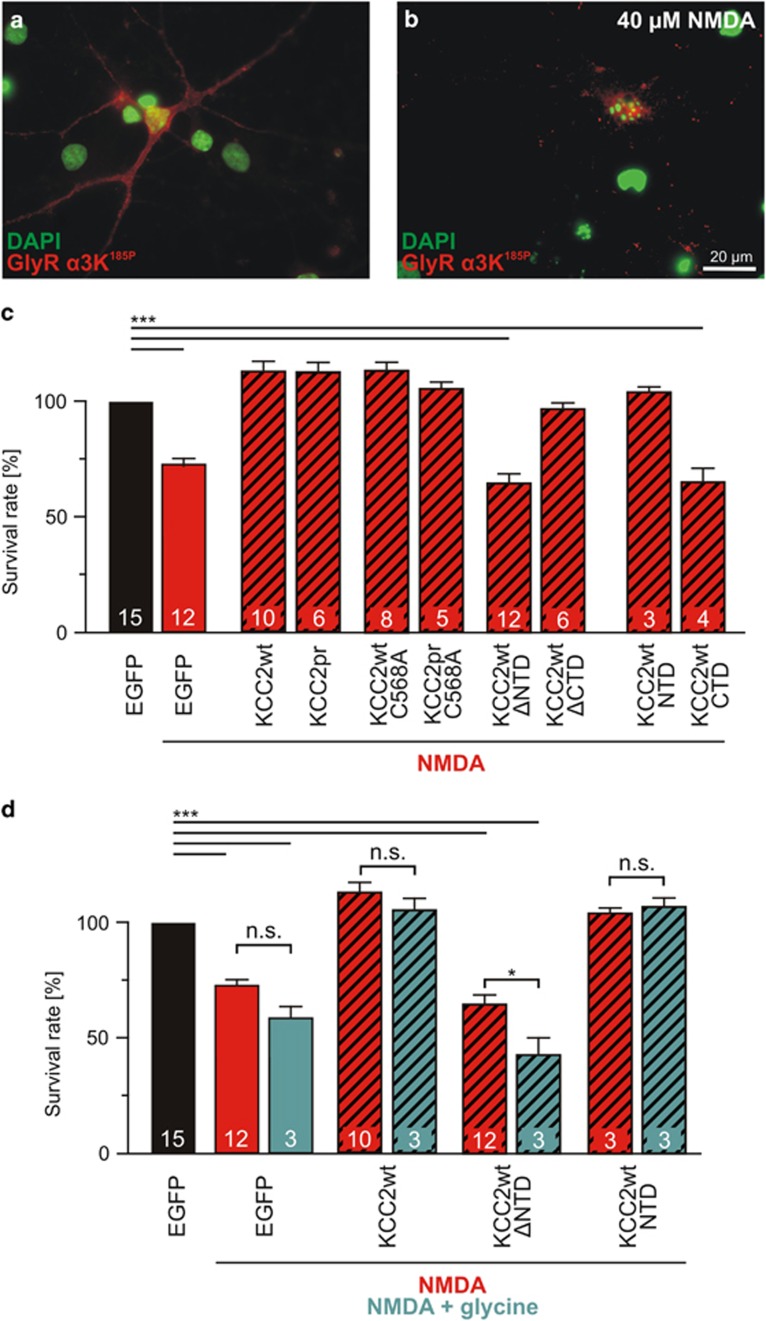
Protein structural aspects of KCC2-dependent neuroprotection in the NMDA model of neurodegeneration. (**a** and **b**) Images of control neurons (**a**) and neurons treated for 30 min with 40 *μ*M NMDA 24 h prior to fixation (**b**) are shown. GlyR *α*3K^185P^ was expressed under non-receptor-activating conditions and surface stained in order to assess neurodegeneration in the same way as in the GlyR-dependent model of neurodegeneration (Figure 1). Degenerated neurons could be identified by their fragmented dendrites and pyknotic nuclei (**b**). (**c**) Quantification of the fraction of surviving neurons revealed that co-expression of KCC2wt or KCC2pr also protects NMDA-treated neurons. Again, Cl^−^ transport-deficient KCC2wt/pr-C568A also rescued neuronal survival. N-terminally truncated KCC2-ΔNTD or the isolated KCC2-CTD was not able to mediate neuroprotection. Note that the isolated KCC2-NTD was sufficient to mediate full rescue of neuronal survival. (**d**) Quantification of the effects of glycine (10 *μ*M) on NMDA-dependent excitotoxicity and KCC2-dependent neuroprotection. Numbers in the bar graphs indicate the number of cultures analyzed. For values, see Supplementary Table 1. Statistical significance is indicated with ***P<0.001

To sustain the finding that KCC2-NTD plays a pivotal role in neuroprotection, we investigated whether neuroprotective function of KCC2-NTD holds in another model of neurodegeneration. In contrast to our GlyR *α*3K^185L^-dependent model of neurodegeneration, which uses chronic silencing of neuronal activity, the NMDA-dependent excitotoxicity model relies on neuronal overexcitation. Actually, KCC2 Cl^−^ transport activity was recently postulated to mediate neuroprotection in the NMDA model of neurodegeneration.^15^ Therefore, we tested KCC2wt and mutant KCC2 variants coupled to EGFP via 2A peptides (Supplementary Figure 1) in the NMDA-dependent model of neurodegeneration.^15^ Neurons were transfected at DIV 6 with non-edited GlyR *α*3K^185P^ (to assess neuronal morphology in addition to appearance of pyknotic nuclei for quantification of neurodegeneration, Figures 7a and b), kept in culture for two days (in non-GlyR-activating conditions), and then incubated for 30 min in 40 *μ*M NMDA in the absence or presence (10 *μ*M) of glycine before maintaining the culture for 24 h as described by Pellegrino *et al.*^15^ Glycine is a co-agonist of NMDARs and therefore is expected to enhance the effect of NMDA with regard to neurodegeneration, but NMDAR internalization^27^ may interfere with this process. However, NMDA effects on neuronal survival in the absence and presence (10 *μ*M) of glycine were comparable, though slightly more pronounced in the presence of glycine (Figures 7c and d; Supplementary Table 1). Co-expression of KCC2wt and KCC2pr protected neurons against NMDA-induced excitotoxicity (Figure 7c), and the Cl^−^ transport-deficient KCC2wt/pr-C568A mutants also succeeded (Figure 7c; Supplementary Table 1). Most importantly, KCC2-ΔNTD and KCC2-CTD failed to protect neurons against NMDA-induced excitotoxicity, whereas KCC2-ΔCTD and KCC2-NTD rescued neuronal survival (Figures 7c and d), similar to our GlyR model of neurodegeneration. Thus, in contrast to previous findings,^15^ our results identify a common mechanism of KCC2-dependent neuroprotection in different models of neurodegeneration, which is independent of Cl^−^ transport activity but involves the KCC2-NTD.

## Discussion

We elucidated the mechanisms of KCC2-dependent neuroprotection and identify a protein structural role of KCC2-NTD in neuroprotection in the glycine-dependent model of chronic neuronal silencing^14^ and the NMDA-dependent model of excitotoxicity.^15^

### Mechanisms of neurodegeneration

Our study ruled out the possibility that inability of chronically silent neurons to become spontaneously activated and participate in spontaneous neural activity in the developing network is a reason for GlyR *α*3K^185L^-dependent neurodegeneration because block of GABA_A_R activation, which provides a major driving force for spontaneous neuronal activity at this developmental stage in the culture dish (Supplementary Figure 2) or action potential-dependent synaptic transmission did not influence survival of neurons with enduring GlyR *α*3K^185L^ activation. Rather, all results synergistically point to enduring GlyR *α*3K^185L^ activation dependent, long-lasting changes of neuronal intrinsic physiological properties involving decreased R_N_, V_rev_ and V_m_ as reasons for GlyR *α*3K^185L^-dependent neurodegeneration. Actually, plasma membrane perforation and resulting impairment of intracellular ion homeostasis were recently associated with the pathophysiology of Alzheimer's disease^28^ but GlyR *α*3K^185L^-dependent neurodegeneration could not be associated with increased intracellular resting [Ca^2+^] as it may be the case in patients with Alzheimer's disease.^28^ This is possibly due to inactivation of, or changes in, expression of voltage-gated Ca^2+^ channels as a consequence of decreased V_m_ in neurons with enduring GlyR *α*3K^185L^ activation.

In the hippocampus, RNA-edited GlyR *α*3K^185L^ contributes to tonic inhibition of cells with low GlyR *β* subunit protein expression.^14, 29, 30, 31, 32, 33, 34^ RNA splicing generates the long variant GlyR *α*3L^185L^, which is preponderantly expressed in the hippocampus and operates at presynaptic sites by contributing to regulation of neurotransmitter release.^12, 29, 35, 36^ However, as the ratio between *α*3K and *α*3L is increased in TLE patients with hippocampal sclerosis (i.e., neurodegeneration),^29^ our results regarding mechanisms of GlyR *α*3K^185L^-dependent neurodegeneration and KCC2-mediated neuroprotection are relevant to the understanding of the pathophysiology of TLE. RNA splicing and its regulatory impact on subcellular distribution of ligand-gated GlyRs and GABA_A_Rs indeed recently emerged as a critical determinant of neuronal dysfunction in TLE,^29, 37, 38, 39^ and non-synaptic Cl^−^ channels have also been implicated by other groups in neuronal cell death. A recent study even provides a link between non-synaptic NMDAR activation and excitotoxicity through Ca^2+^-activated Cl^−^ channel Clca1.^18^ With regard to neurodegeneration, alteration of non-synaptic Cl^−^ channel function thus represents an important determinant of cellular programs that elicit cell death, and for this reason, care should be taken when considering the use of glycine for neuroprotective purposes.^40, 41, 42^

### Identification of the neuroprotective capacity of the KCC2-NTD in different models of neurodegeneration

KCC2 is a developmentally regulated gene product, which can undergo functional downregulation in the diseased brain.^7, 8, 9, 43, 44^ Functional downregulation involves phosphorylation of amino acids in the KCC2-CTD.^24, 25^ In our study, KCC2wt-dependent Cl^−^ extrusion was apparent only in cortical neurons (Supplementary Figure 5), which demonstrates functionality of KCC2wt-dependent Cl^−^ extrusion in general, but also identifies neuron type-specific (cortical *versus* hippocampal neurons) differences in the apparent efficacy of Cl^−^ extrusion through KCC2wt. The discrepancy to other studies in which KCC2wt overexpression was reported to significantly shift E_GABA_ to hyperpolarized potentials in cultured hippocampal neurons (e.g., Li *et al.*^5^and Chudotvorova *et al.*^45^) is probably due to differences in the cell culture preparation (e.g., cell density, duration and strength of KCC2 expression, time point of investigation and percentage of GABAergic interneurons in the culture dish). However, hippocampal neurons in our culture preparations develop normally as they are excitable and spontaneously active due to depolarizing GABA_A_R signaling (Supplementary Figure 2). Thus, the discrepancy to other studies might involve culture-specific changes in the phosphorylation of the S940 site, which was shown to enhance Cl^−^ extrusion capacity of KCC2wt.^46, 47^ Nevertheless, as Cl^−^ extrusion through the KCC2pr variant (which cannot be phosphorylated at Y903, T906, T1007 and Y1087) was apparent in our hippocampal neuron preparations (Supplementary Figures 4 and 5, and Figure 5d), our results clearly identify a role for these sites in phosphorylation-dependent regulation of KCC2 Cl^−^ extrusion, which is in agreement with mounting evidence for the role of threonine phosphorylation in the downregulation of KCC2-dependent Cl^−^ transport (for review see Kahle *et al.*^48^). Our results furthermore make a clarifying contribution to the controversial discussion of the role of tyrosine phosphorylation in the regulation of KCC2 Cl^−^ extrusion capacity^48^ as they show that KCC2pr with unphosphorylatable threonine and tyrosine residues has an increased Cl^−^ extrusion capacity. However, more detailed study is necessary to fully clarify the role of threonine and tyrosine phosphorylation.

Beyond its ‘classical' function as Cl^−^ transporter that contributes to the developmental switch of GABA action from de- to hyperpolarization,^3^ KCC2 fulfills a protein structural function, which contributes to co-regulation of glutamatergic and GABAergic synapses during synaptogenesis in development.^4, 5^ The neuroprotective effect of KCC2 was demonstrated already in 2008,^14^ but whether KCC2-dependent neuroprotection involves a protein structural function or regulation of intracellular [Cl^−^] is a matter of debate.^11, 14, 15^ Although our initial study with GlyR-dependent neuronal hypoactivity-induced neurodegeneration^14^ did not address this question, a follow-up study showed that KCC2-dependent neuroprotection depends on its Cl^−^ transport activity in the NMDA-dependent hyperexcitation model of neurodegeneration.^15^

**Figure 8:**
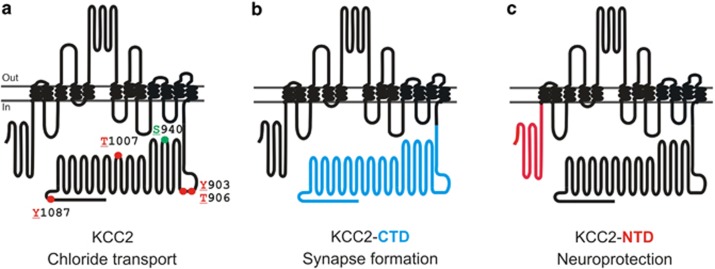
Different functional roles of KCC2. The scheme summarizes previous results and findings presented here. (**a**) Full-length KCC2 is a Cl^−^ transporter that can be positively (S940; green) or negatively (Y903, T906, T1007 and Y1087; red, this study) regulated by phosphorylation. (**b**) The synaptogenic function of the KCC2-CTD (blue) is established and described elsewhere.^5, 26^ (**c**) Neuroprotective function of the isolated KCC2 N-terminal domain (KCC2-NTD, this study)

By challenging primary hippocampal neurons with these two different experimental neurotoxic strategies including enduring GlyR *α*3K^185L^ activation associated with chronic inhibition of neuronal activity as it may occur in the hippocampus of TLE patients^14^ and NMDA-dependent excitotoxicity,^15^ our study provides compelling evidence for a protein structural role of KCC2 in neuroprotection. This conclusion is based on the fact that both KCC2wt (which did not effectively show Cl^−^ extrusion in primary hippocampal neurons; Supplementary Figures 4 and 5, and Figures 5d and e) and Cl^−^ transport-deficient KCC2-C568A rescued neuronal survival in both neurotoxic conditions. Most strikingly, the KCC2-NTD was neuroprotective in both experimental models of neurodegeneration. All these results provide evidence for a protein structural neuroprotective role of KCC2 in neuroprotection, and consistently, our study identifies the KCC2-NTD as the relevant KCC2 protein domain sufficient for neuroprotection. In contrast to the rather large (299 amino acid encompassing) KCC2-CTD with its well-established protein structural role in synapse formation,^4, 5^ ectopic expression of the relatively compact, 100 amino acid spanning, KCC2-NTD (Supplementary Figure 1A) is a novel suitable but non-exclusive strategy to prevent neurodegeneration in TLE and other neurodegenerative diseases as it would work independently of the KCC2-CTD (involved in regulation of synapse formation) or full-length KCC2-dependent regulation of chloride transport. Thus, KCC2 is a moonlighting protein^49^ as it is not only a protein with Cl^−^ transport activity but also harbors several protein domains with synaptogenic and neuroprotective activities (Figure 8).

## Materials and Methods

### Molecular cloning

A cDNA coding for hKCC2b wildtype (designated ‘wt') was kindly provided by the laboratory of Kai Kaila (Helsinki, Finland) and recently used in one of our studies.^14^ The KCC2a cDNA was cloned from human postmortem hippocampus RNA (pool of 20 healthy Caucasians, Clontech (Palo Alto, CA, USA). By using the 2A-self-processing peptide EGRGSLLTCGDVEENPGP derived from Thosea asigna,^50^ we generated constructs for expression of KCC2 and mCherry or EGFP from polycistronic mRNA (Supplementary Figure 1). To generate the phosphorylation-resistant variant KCC2pr, which cannot be functionally downregulated, we substituted by site-directed mutagenesis (GeneEditor, Promega, Mannheim, Germany) alanine and phenylalanine for threonine and tyrosine, respectively, at the relevant positions in the CTD;^24, 25^ see Supplementary Figure 1 for sequence details. The amino acid substitution C568A was generated using QuickChange Lightning site-directed mutagenesis kit (Agilent Technologies, Waldbronn, Germany). Truncations were generated using the full-length KCC2 constructs and appropriate primer sets. All the KCC2 constructs for neuronal expression are equipped with a cytomegalovirus (CMV) enhancer-human synapsin-1 promoter (hSyn1). All the constructs contain the woodchuck posttranscriptional regulatory element (WPRE), which was derived from a lentiviral vector (Clontech). All expression constructs were verified with DNA sequencing. Molecular cloning of GlyR *α*3K^185P^ and GlyR *α*3K^185L^ constructs is described in former publications of our laboratory.^10^

### Cell culture and transfection

All animals were killed according to the permit given by the Office for Health Protection and Technical Safety of the regional government of Berlin (LaGeSo, 0122/07) and in compliance with regulations laid down in the European Community Council Directive. Hippocampal cells were isolated from E19 Wistar rats and kept in B27-supplemented Neurobasal medium (Life Technologies) in 24-well plates as described.^14^ Transfection was carried out on DIV 6 using 300 ng DNA per well in combination with Effectene transfection reagent (Qiagen, Hilden, Germany), following the manufacturer's protocol as described.^14^ Either low (185P) or high (185L) affinity receptor types of the short (K) splice variant of GlyR *α*3^10^ were expressed for the duration of 3 days in the presence or absence of added glycine (0, 10 or 400 *μ*M). For this purpose, a glycine-free minimal essential medium was used.^14^ In some experiments, neurons were exposed to NMDA (40 *μ*M) for 30 min in the absence or presence of 10 *μ*M glycine and analyzed 24 h after application. For co-transfection, 60 ng of KCC2-coding plasmids were mixed with 240 ng GlyR *α*3K^185L^. For Oregon Green imaging of GABA-elicited Ca^2+^ signals, neurons were investigated at an earlier time point in cell culture, at DIV 6–7.

### Chemicals

HEPES (4-(2-Hydroxyethyl)piperazine-1-ethanesulfonic acid) and all inorganic salts as well as glycine (G7126), GABA (A2129), NMDA (M3262), gramicidin (G5002), lucifer yellow (L0144) and bicuculline methiodide (14343) were purchased from Sigma-Aldrich (Steinheim, Germany). GABAzine (1262) came from Tocris (Bristol, UK), TTX (6973.1) from Carl Roth (Karlsruhe, Germany), 4-bromo-A-23187 (BML-CA101-0001) and ionomycin (LKT-I5753-M001) from ENZO Life Sciences (Lörrach, Germany). Fura-2/AM and Oregon Green 488/AM were purchased from Life Technologies. Stock solutions were made with H_2_O in the case of TTX (1 mM), glycine and GABA (1 M each), and with DMSO (dimethyl sulfoxide) (SERVA Electrophoresis GmbH, Heidelberg, Germany) in the case of gramicidin, 4-bromo-A-23187 and ionomycin (10 mM each).

### Immunochemistry

A polyclonal chicken antibody was used to visualize HA-tagged surface GlyR (1 : 200, Bethyl Laboratories, Montgomery, TX, USA). The secondary antibody was made in donkey, conjugated with indocarbocyanine (Cy3) and purchased from Jackson ImmunoResearch Laboratories (Suffolk, UK). Surface staining of HA-tagged GlyR *α*3K was performed for 5 min at 37 °C in cell culture medium, as described.^14, 29^ Prior to fixation with paraformaldehyde (PFA) cells were washed three times with cell culture medium. For fixation with PFA, cells were incubated for 15 min at room temperature (RT) with ice-cold PBS containing 4% PFA and 4% sucrose, followed by three wash steps in PBS. Incubation of fixed cells in freshly made 50 mM NH_4_Cl for 15 min at RT was used to quench free aldehyde groups from the PFA fixation. Cells were then again washed three times in PBS and blocked with PBS/gelatine (0.1%) prior to permeabilization with 0.12% Triton X-100(Sigma-Aldrich) in PBS/gelatine for 4 min at RT. Before incubation with first antibodies, coverslips were again washed with PBS/gelatine. In some experiments, KCC2 was stained for 1 h at RT using a rabbit polyclonal antibody (#07-432; Merck Millipore, Germany) diluted 1 : 200 in PBS/gelatine. Prior to incubation with secondary antibodies cells were washed three times with PBS/gelatine. For KCC2 stainings, a secondary antibody made in donkey and conjugated with indocarbocyanine (Cy5, Jackson ImmunoResearch Laboratories) was used. After 45 min of incubation at RT cells were again washed three times with PBS/gelatine followed by additional two wash steps in PBS. Stained cell preparations were finally mounted on microscope slides using DAPI-containing vectashield medium (Vector Laboratories, Peterborough, UK).

### Electrophysiology

An EPC-7 amplifier and Patchmaster software (HEKA, Lambrecht, Germany) were used for patch-clamp recordings. Patch pipettes, made from borosilicate glass (Science Products, Hofheim, Germany), had resistances of 2–6 MΩ when filled with the intracellular solution containing (in mM) KCl (130), NaCl (5), CaCl_2_ (0.5), MgCl_2_ (1), EGTA (5) and HEPES (30). Application of substances was gravity driven. The tip (360 *μ*M) of a perfusion pencil (AutoMate Scientific, Berkeley, CA, USA) was placed close (ca 100 *μ*M) to the recorded neuron to ensure relatively rapid application of substances. Under these conditions, the wash-in duration of lucifer yellow-containing test solution was <500 ms. For the perforation of the patch, the pipette solution contained additionally 50–100 *μ*M gramicidin and 100 *μ*M lucifer yellow, which allowed monitoring the stability of the perforated patch (Supplementary Figure 3A). In addition, a strong shift of the baseline current due to the high Cl^−^ concentration in the pipette solution indicated membrane rupture upon transition to whole-cell configuration. In this case, the recording was stopped. The standard extracellular solution (E1; pH 7.4) contained (in mM) NaCl (140), KCl (5), MgCl_2_ (1), CaCl_2_ (2), HEPES-NaOH (10) and glucose (10). In the voltage-clamp mode, neurons were clamped at a potential of −50 mV. IV relationships were obtained from voltage ramps from −100 to −30 mV with a duration of 140 ms applied every 5 s. Series and input resistances were checked throughout the whole duration of each experiment by applying −5 mV pulses prior to the voltage ramps. All data were acquired with a sampling rate of 10 kHz after filtering at 2.8 kHz. All experiments were performed at RT (20–25 °C).

### Calcium imaging

Prior to ratiometric and non-ratiometric Ca^2+^ imaging experiments cells were loaded with fura-2/AM or Oregon Green 488/AM, respectively, by incubating the cells in E1 buffer (see above) supplemented with 1–5 *μ*M of fura-2 or Oregon Green for 20 min at 37 °C. Subsequently, cells were incubated for further 20 min in E1 to ensure deesterification. Glass coverslips with the dye-loaded neurons were placed into a recording chamber (ca 1 ml volume) on the stage of an Axiovert 10 or an Axio Lab.A1 micoroscope (both Zeiss, Oberkochen, Germany). Cells were submerged with a constant flow of E1 through an infusion pipette, which was placed in close vicinity (ca 200 *μ*m) to the recorded cells to ensure short wash-in/washout durations. Transfected cells were identified by the fluorescence of mCherry, which served as control or was co-expressed using the 2A-self-cleaving peptide in KCC2 constructs (see Supplementary Figure 1B for constructs). Ratiometric measurements were performed with the Polychrome V, a Clara Interline CCD camera (Andor Technology, Belfast, UK) and Live Acquisition software (Till Photonics, Martinsried, Germany) using 340 and 380-nm excitation wavelengths. Excitation and emission light were separated by a 510-nm dichroic mirror. The emitted light was filtered using a 530-nm longpass filter. Exposure times were 20 ms (340 nm) and 5 ms (380 nm), and the rate for [Ca^2+^] measurement was set to one pair of images per 1 sec. 50 mM KCl were applied to check the viability of the cells. To obtain minimal (0 mM) and maximal (10 mM) Ca^2+^ signals for calibration,^20^ cells were permeabilized with either 10 *μ*M ionomycin or 10 *μ*M 4-bromo-antibiotic A23187. 2 mM Mn^2+^ were applied at the end of each experiment to quench the signal and thus to obtain background fluorescence that was subtracted from all F340 and F380 values used for [Ca^2+^]_i_ calculation according to:


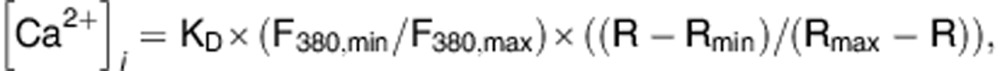


where K_D_ is the dissociation constant of the fura-2-Ca^2+^complex (225 nM^51^). F_380,min_ and F_380,max_ are the fluorescence values of free (0 mM, min) and Ca^2+^-bound (10 mM, max) fura-2. R, R_min_, R_max_ are the fluorescence ratios (F_340_/F_380_) at the beginning of the experiment (R) and after permeabilization in 0 mM (R_min_) and 10 mM (R_max_) Ca^2+^, respectively. Non-ratiometric experiments were performed with an HBO-100 lamp (OSRAM GmbH, Munich, Germany), an electronic shutter, a SPOT pursuit camera and Metamorph software (Visitron Systems, Puchheim, Germany). Oregon Green fluorescence was excited and detected using an appropriate filter set (XF22, Omega Opticals, Olching, Germany) using 100 ms shutter open times at a frequency of three per second. Oregon Green fluorescence signals are expressed as F_GABA_/F_KCl_, where F_KCl_ and F_GABA_ are the fluorescence values in the presence of E1 supplemented with 50 mM KCl (90 mM instead of 140 mM NaCl) and 100 *μ*M GABA, respectively.

### Data analysis and statistics

All numerical data are reported as mean±S.E.M. Statistical analysis (ANOVA and *post hoc* Tukey's test) was performed using the software IGOR Pro 6.3 (WaveMetrics, Lake Oswego, OR, USA). Significance levels are indicated as *P<0.05, **P<0.01 and ***P<0.001.

Quantitative data of neuronal survival is presented as values that are normalized to the number of vital neurons in control conditions. Degenerated and vital neurons were counted from at least three independent hippocampal cell cultures. The number of experiments is indicated in brackets in the bar graphs. The conditions GlyR *α*3K^185P^ and *α*3K^185L^ in the presence of 10 *μ*M glycine were included in each experiment for normalization purpose.

V_rev_ was calculated by determining the voltage at which no net current was observed (zero point of the basal IV curve in the absence of GABA, IV_bas_). The values presented in figures and text are not corrected for the liquid junction potential (+3.75 mV). E_GABA_ was obtained by determining the zero point of the GABA IV relationship that was calculated by subtraction of IV_bas_ from IV_GABA_ (Figure 4). Analysis was performed by a homemade procedure written using IGOR Pro 6.3 (WaveMetrics).

## Abbreviations

CMV: cytomegalovirus
CTD: C-terminal KCC2 protein domain
EGABA: V_rev_ of GABAAR-dependent currents
GABA: gamma-aminobutyric acid
GABAAR: GABA type A receptor
GlyR: glycine receptor
HEPES: 4-(2-hydroxyethyl)-1-piperazineethanesulfonic acid
hSyn1: human synapsin-1
IV relationships: current-voltage relationships
KCC2: potassium chloride co-transporter 2
KCC2pr: phosphorylation-resistant KCC2 variant
NKCC1: sodium potassium chloride co-transporter 1
NTD: N-terminal KCC2 protein domain
NMDA: N-methyl-D-aspartate
NMDARs: NMDA receptors
PBS: phosphate-buffered saline
PFA: paraformaldehyde
RN: membrane resistance
TLE: temporal lobe epilepsy
TTX: tetrodotoxin
V_rev_: membrane reversal potential
WPRE: woodchuck posttranscriptional regulatory element
