# Supplementary material for: Chloride transporter KCC2-dependent neuroprotection depends on the N-terminal protein domain
Source: Cell Death Dis. 2015 Jun 4;6(6):e1776–. doi: 10.1038/cddis.2015.127 (PMC4669822; doi:10.1038/cddis.2015.127)
Supplement: Supplementary Information [file cddis2015127x1.doc]

**Supplemental Information**

**Chloride transporter KCC2-dependent neuroprotection depends on the
N-terminal protein domain**

Aline Winkelmann1*, Marcus Semtner1* and Jochen C. Meier1,2

1 RNA editing and Hyperexcitability Disorders Helmholtz Group, Max Delbrück Center for Molecular Medicine, 13125 Berlin, Germany

2 TU Braunschweig, Zoological Institute, Division of Cell Biology & Cell Physiology, 38106 Braunschweig, Germany

* Equal contribution

**Correspondence:**

Drs. Marcus Semtner and Jochen C. Meier

Max Delbrück Center for Molecular Medicine

Robert-Rössle-Straße 10, 13125 Berlin

Germany

Phone: +49-(0)30-9406-3062/3700

Email: marcus.semtner@mdc-berlin.de, jochen.meier@mdc-berlin.de

**Short title:** Mechanisms of KCC2-dependent neuroprotection

**Suppl. Table 1**

Overview over the transfections, medium conditions, survival rates (% vital), number of experiments [N (HC)] and analyzed neurons [N (neurons)], and significant differences between control (α3K185P, normalized to 100%) and the different experimental conditions (*P*). *P* values are the results from posthoc Tukey test (after ANOVA1) of all values of the respective figure. Neurodegenerative conditions are colored red, and experimental conditions that rescued neuronal survival are colored green.

| **Transfection** | **Medium**  **supplements** | **% vital** | **SEM** | **N**  **(HC)** | **N (neurons)** | ***p*** |
| --- | --- | --- | --- | --- | --- | --- |
| **Fig. 1C** | | | | | | |
| GlyR α3K185P | 10 µM Gly | 100 | 0.0 | 84 | 15428 | - |
| GlyR α3K185L | 10 µM Gly | 55.2 | 1.4 | 79 | 10588 | <0.001 |
| GlyR α3K185P | 400 µM Gly | 30.3 | 3.6 | 7 | 576 | <0.001 |
| GlyR α3K185L | 0 Gly | 87.3 | 6.2 | 3 | 551 | 0.0759 |
| GlyR α3K185L  KCC2a | 10 µM Gly | 100.8 | 3.1 | 3 | 281 | 0.9997 |
| GlyR α3K185L  KCC2b | 10 µM Gly | 99.1 | 2.7 | 45 | 4251 | 0.9744 |
| **Fig. 2A** | | | | | | |
| GlyR α3K185P | 10 µM Gly | 100 | 0.0 | 84 | 15428 | - |
| GlyR α3K185L | 10 µM Gly | 55.2 | 1.4 | 79 | 10588 | <0.001 |
| GlyR α3K185L | 10 µM Gly  0.2 µM GABAzine | 60.1 | 7.7 | 4 | 414 | <0.001 |
| GlyR α3K185L | 10 µM Gly +  3 µM GABAzine | 57.2 | 6.7 | 3 | 315 | <0.001 |
| GlyR α3K185L  KCC2wt | 10 µM Gly | 99.1 | 2.7 | 45 | 4251 | 0.9996 |
| GlyR α3K185L  KCC2wt | 10 µM Gly +  0.2 µM GABAzine | 100.7 | 6.2 | 3 | 329 | 0.9999 |
| GlyR α3K185L  KCC2wt | 10 µM Gly +  3 µM GABAzine | 103.5 | 3.9 | 3 | 407 | 0.9987 |
| **Fig. 2B** | | | | | | |
| GlyR α3K185P | 10 µM Gly | 100 | 0.0 | 84 | 15428 | - |
| GlyR α3K185P | 10µM Gly +  1 µM TTX | 95.7 | 10.4 | 6 | 540 | 0.9630 |
| GlyR α3K185L | 10 µM Gly | 55.2 | 1.4 | 79 | 10588 | <0.001 |
| GlyR α3K185L | 10µM Gly +  1 µM TTX | 56.7 | 7.8 | 6 | 351 | <0.001 |
| GlyR α3K185L  KCC2wt | 10 µM Gly | 99.1 | 2.7 | 45 | 4251 | 0.9744 |
| GlyR α3K185L  KCC2wt | 10µM Gly +  1 µM TTX | 89.7 | 8.9 | 3 | 244 | 0.7192 |
| **Fig. 6A** | | | | | | |
| GlyR α3K185P | 10 µM Gly | 100 | 0 | 84 | 15428 | - |
| GlyR α3K185P  Bumetanide | 10 µM Gly | 96.7 | 2.2 | 4 | 552 | 0.8862 |
| GlyR α3K185L | 10 µM Gly | 55.2 | 1.4 | 79 | 10588 | <0.001 |
| GlyR α3K185L  Bumetanide | 10 µM Gly | 62.5 | 7.5 | 4 | 273 | <0.001 |
| **Fig. 6B** | | | | | | |
| GlyR α3K185P | 10 µM Gly | 100 | 0.0 | 84 | 15428 | - |
| GlyR α3K185L | 10 µM Gly | 55.2 | 1.4 | 79 | 10588 | <0.001 |
| GlyR α3K185L  KCC2wt | 10 µM Gly | 99.1 | 2.7 | 45 | 4251 | 0.9984 |
| GlyR α3K185L  KCC2pr | 10 µM Gly | 97.7 | 4.1 | 12 | 584 | 0.9882 |
| GlyR α3K185L KCC2wt-C568A | 10 µM Gly | 92.6 | 4.7 | 6 | 194 | 0.6664 |
| GlyR α3K185L KCC2pr-C568A | 10 µM Gly | 96.6 | 2.9 | 6 | 183 | 0.9826 |
| GlyR α3K185L KCC2wt-ΔNTD | 10 µM Gly | 75.1 | 4.2 | 7 | 220 | <0.001 |
| GlyR α3K185L  KCC2wt-ΔCTD | 10 µM Gly | 97.7 | 5.4 | 4 | 603 | 0.9997 |
| GlyR α3K185L  KCC2wt-NTD | 10 µM Gly | 111.0 | 5.1 | 3 | 797 | 0.6802 |
| GlyR α3K185L KCC2wt-CTD | 10 µM Gly | 73.8 | 7.8 | 5 | 264 | <0.001 |
| **Fig. 7C** | | | | | | |
| GlyR α3K185P | 0 µM Gly | 100 | 0.0 | 15 | 3947 | - |
| GlyR α3K185P | 0 µM Gly  40 µM NMDA | 72.7 | 2.7 | 12 | 2834 | <0.001 |
| GlyR α3K185P KCC2wt | 0 µM Gly  40 µM NMDA | 112.6 | 4.2 | 10 | 905 | 0.0574 |
| GlyR α3K185P KCC2pr | 0 µM Gly  40 µM NMDA | 112.0 | 4.6 | 6 | 472 | 0.2582 |
| GlyR α3K185P KCC2wt-C568A | 0 µM Gly  40 µM NMDA | 112.7 | 3.3 | 8 | 938 | 0.0721 |
| GlyR α3K185P KCC2pr-C568A | 0 µM Gly  40 µM NMDA | 106.3 | 2.8 | 5 | 387 | 0.9592 |
| GlyR α3K185P KCC2wt-ΔNTD | 0 µM Gly  40 µM NMDA | 65.3 | 4.0 | 12 | 933 | <0.001 |
| GlyR α3K185P KCC2wt-ΔCTD | 0 µM Gly  40 µM NMDA | 97.4 | 2.5 | 6 | 1327 | 0.9999 |
| GlyR α3K185P KCC2wt-NTD | 0 µM Gly  40 µM NMDA | 104.6 | 2.2 | 3 | 507 | 0.9989 |
| GlyR α3K185P KCC2wt-CTD | 0 µM Gly  40 µM NMDA | 65.8 | 5.8 | 4 | 608 | 0.0011 |
| **Fig. 7D** | | | | | | |
| GlyR α3K185P | 0 µM Gly | 100 | 0.0 | 15 | 3947 | - |
| GlyR α3K185P | 0 µM Gly  40 µM NMDA | 72.7 | 2.7 | 12 | 2834 | <0.001 |
| GlyR α3K185P | 10 µM Gly  40 µM NMDA | 58.3 | 5.0 | 3 | 959 | <0.001 |
| GlyR α3K185P KCC2wt | 0 µM Gly  40 µM NMDA | 112.6 | 4.2 | 10 | 905 | 0.0615 |
| GlyR α3K185P KCC2wt | 10 µM Gly  40 µM NMDA | 106.3 | 5.1 | 3 | 704 | 0.9851 |
| GlyR α3K185P KCC2wt-ΔNTD | 0 µM Gly  40 µM NMDA | 65.3 | 4.0 | 12 | 933 | <0.001 |
| GlyR α3K185P KCC2wt-ΔNTD | 10 µM Gly  40 µM NMDA | 41.9 | 7.5 | 3 | 444 | <0.001 |
| GlyR α3K185P KCC2wt-NTD | 0 µM Gly  40 µM NMDA | 104.6 | 2.2 | 3 | 507 | 0.9979 |
| GlyR α3K185P KCC2wt-NTD | 10 µM Gly  40 µM NMDA | 107.7 | 3.8 | 3 | 472 | 0.9472 |

**Suppl. Fig. 1: KCC2 protein structure.** (A) Membrane topology of KCC2. The N- (red) and C-terminal (blue) domains, Exon1 (green), the location of C568 as well as the KCC2wt threonine and tyrosine phosphorylation sites (red dots) are illustrated. The position of the S940 phosphorylation site in the C-terminal KCC2 domain sequence that is involved in up-regulation of KCC2 Cl- extrusion capacity is indicated in green color. Protein sequences corresponding to the transmembrane domains 9-12 are shown underlined and light blue. (B) Scheme illustrating our KCC2 expression constructs. KCC2 was coupled either to EGFP or to mCherry via 2A self-processing peptides. Furthermore, constructs contained the woodchuck posttranscriptional regulatory element (WPRE) which was derived from a lentiviral vector (Clontech).

**Suppl. Fig. 2: Sponteanous postsynaptic currents (sPSCs) of DIV6-7 primary hippocampal neurons.** (A) DIC image of a recorded hippocampal neuron. The bath application was placed close to (at 100 µm distance to) the recorded neurons to reduce wash-in duration and artifacts. (B) An increase of the extracellular K+ concentration from 5 mM to 15 mM leads to a depolarizing shift of the basal membrane current (at -50 mV holding potential) and a strong increase in sPSC frequency. (C) Bicuculline (20 µM) and 10 µM DNQX as well as 50 µM APV were used to block GABAARs and AMPARs/NMDARs, respectively. Representative traces show the effects of bicuculline and DNQX/APV on sPSCs in the presence of 15 mM KCl. (D-F) Quantification of basal whole-cell currents (-50 mV, D), input resistances (E) and sPSC frequencies (F) in 5 mM and 15 mM extracellular KCl. Note that sPSCs at DIV6-7 are mediated by GABAARs and AMPARs/NMDARs, but bicuculline alone sufficed to largely prevent the 15 mM KCl-dependent increase in sPSC frequency (F). Numbers in the bar graphs indicate the number of neurons analyzed. Statistical significance is indicated with *: *P* < 0.05.

**Suppl. Fig. 3: Electrophysiological properties of GlyR α3K185L-expressing neurons.** (A)The images illustrate the perforated patch clamp procedure. Transfected neurons were identified by their mCherry fluorescence (*right top*). Pipette solution contained 50‑100 µM gramicidin and 100 µM lucifer yellow to monitor the stability of the perforated patch (*left bottom*). When the patch was disrupted, Lucifer yellow diffused into the soma (*right bottom*) and the recording configuration switched to the whole-cell mode. In this case, the recording was stopped. (B) Primary hippocampal neurons were either transfected with mCherry alone (Control) or with mCherry and GlyR α3K185L (185L) and cultured for 2-3 days under receptor-activating conditions (10 µM glycine). Neurons were investigated by perforated patch clamp (Gramicidin) and voltage-clamped at different holding potentials (‑80 mV to ‑50 mV). In control neurons at 10 µM glycine, the application of 100 µM GABA lead to strong hyper- and de-polarizing currents depending on the holding potential (B, *left*). In contrast, GABA-evoked currents in GlyR α3K185L-expressing neurons were strongly diminished at all potentials tested (B, *right, red*), indicating the large shunt elicited by GlyR α3K185L activation. Indeed, in nominally glycine-free recording solution (wash-out), GABA-evoked currents also appeared in GlyR α3K185L-expressing neurons (B, *right, light red*). (C) Vm of GlyR α3K185L-expressing neurons at 10 µM glycine was determined 1‑3 min after the switch from the voltage (VC) to the current-clamp (CC) configuration, since membrane potentials slowly depolarized under these conditions from values around ‑50 mV (the former holding potential) to final values of ‑35.9 ± 2.6 mV (n = 5) when the voltage clamp at ‑50 mV was withdrawn (C, red curve). In contrast, Vm of control cells (black curve) was significantly more negative and relatively constant. What could be the reason for different values of Vm (current clamp) and Vrev (voltage clamp; e.g. Figure 4)? Neuronal membranes are not passive and may therefore reveal presence of a couple of voltage-activated conductances which are fixed by the holding potential in the voltage clamp mode but can “equilibrate” when current-clamped at 0 pA. Indeed, the Vrev (and EGABA) of GlyR α3K185L-expressing neurons was clamped towards the imposed holding potential (‑50 mV) in the voltage clamp configuration due to the large Cl- conductance. When the voltage clamp was released, resting membrane potential (Vm) was established according to all the different conductances in the neuronal plasma membrane. The slow depolarization of Vm in GlyR α3K185L-expressing neurons after switching into current clamp indicates on the one hand the large GlyR α3K185L conductance of those neurons and can therefore, on the other hand, be considered as the equilibration of intracellular Cl- through GlyR α3K185L channels; please note here that intracellular Cl- is NOT fixed by the pipette solution in all these experiments as they were gramicidin-perforated measurements. (D) Summary of the resting membrane potentials (Vm) obtained in perforated patch clamp experiments in the current clamp configuration (at 0 pA). Numbers in the bar graphs indicate the number of neurons analyzed. Statistical significance is indicated with *: *P* < 0.05, **: *P* < 0.01.

**Suppl. Fig. 4: Cl- transport activity of KCC2wt and KCC2pr in hippocampal neurons.** (A) Primary hippocampal neurons were either transfected with mCherry (Control), KCC2wt-2A-mCherry or KCC2pr-2A-mCherry and investigated by perforated patch clamp (gramicidin) in the voltage-clamp configuration (holding potential: ‑50 mV). The application of 100 µM GABA lead to hyperpolarizing currents in most of the control and KCC2-expressing neurons (*top*, time points for determination of IVbasal and IVGABA are indicated in current traces). Current-voltage relationships (IV) were obtained by applying every 5 s voltage ramps ranging from ‑100 mV to ‑30 mV. The IVs of GABA-evoked currents (EGABA) were determined by subtracting the IVs in the presence and absence of GABA. (B) Quantification of EGABA revealed that only KCC2pr, not KCC2wt, was able to shift neuronal EGABA significantly to more hyperpolarized values. Numbers in the bar graphs indicate the number of neurons analyzed. Statistical significance is indicated with *: *P* < 0.05, **: *P* < 0.01.

**Suppl. Fig. 5:** **Non-ratiometric Ca2+ imaging with Oregon green (OG488) in primary neurons.** (A) Oregon green (OG488) fluorescence was used to quantify the effects of KCC2 expression on GABA-elicited Ca2+ signals. (A, *top*) Schematic representation of the constructs illustrates that mCherry, KCC2wt-2A-mCherry or KCC2pr-2A-mCherry are flanked by the human synapsin-1 promoter (hSyn1) and the Woodchuck posttranscriptional regulatory element (WPRE). The red dots indicate positions of the threonine and tyrosine residues that were mutated in the KCC2pr construct. (A, *bottom*) Representative images of a view field centered on a transfected cortical neuron are shown. Transfected neurons were identified according to mCherry fluorescence (boxed in red color) and analyzed at DIV6-7 simultaneously with neighboring untransfected cells (boxed in grey color). Oregon green signals in response to GABA (100 µM) or KCl (50 mM) application are shown. Representative traces (B) show alterations in F/F0 in response to application of GABA or KCl in untransfected cells (grey) and in a KCC2wt-positive cortical neuron (red). GABA application led to Ca2+ elevations in untransfected cells but not in the KCC2wt-expressing neurons, indicating a KCC2-dependent shift in EGABA. (C and D) Quantification of GABA-dependent Ca2+ signals normalized to KCl-dependent Ca2+ signals (FGABA/FKCl) measured in hippocampal (C) or cortical neurons (D). Black and hatched bars identify transfected neurons, while grey bars identify neighboring non-transfected neurons. Note that only KCC2pr decreased GABA-mediated Ca2+ elevations in both hippocampal and cortical neurons, whereas KCC2wt was effective only in cortical neurons, which confirms its Cl-­ transport activity and reveals neuron type-specific and phosphorylation-dependent differences in the regulation of Cl- transport through KCC2. Numbers in the bar graphs indicate the number of neurons analyzed. Statistical significance is indicated with **: *P* < 0.01, ***: *P* < 0.001.

**Suppl. Fig. 6:** **Qualitative immunochemical analysis of** **the** **protein expression of KCC2 variants.** Images of neurons co-transfected with GlyR α3K185P and KCC2wt-2A-EGFP, KCC2wt-ΔNTD-2A-EGFP, and KCC2wt-CTD-2A-EGFP are shown. HA-epitope tagged and surface-stained GlyR α3K185P (red), DAPI (blue) and EGFP (green) are shown in top panels. KCC2 was stained using a rabbit polyclonal antibody which binds to the C-terminus of KCC2. Bottom panels show corresponding immunofluorescent signals obtained with this KCC2-antibody. Scale bar: 20 µm.


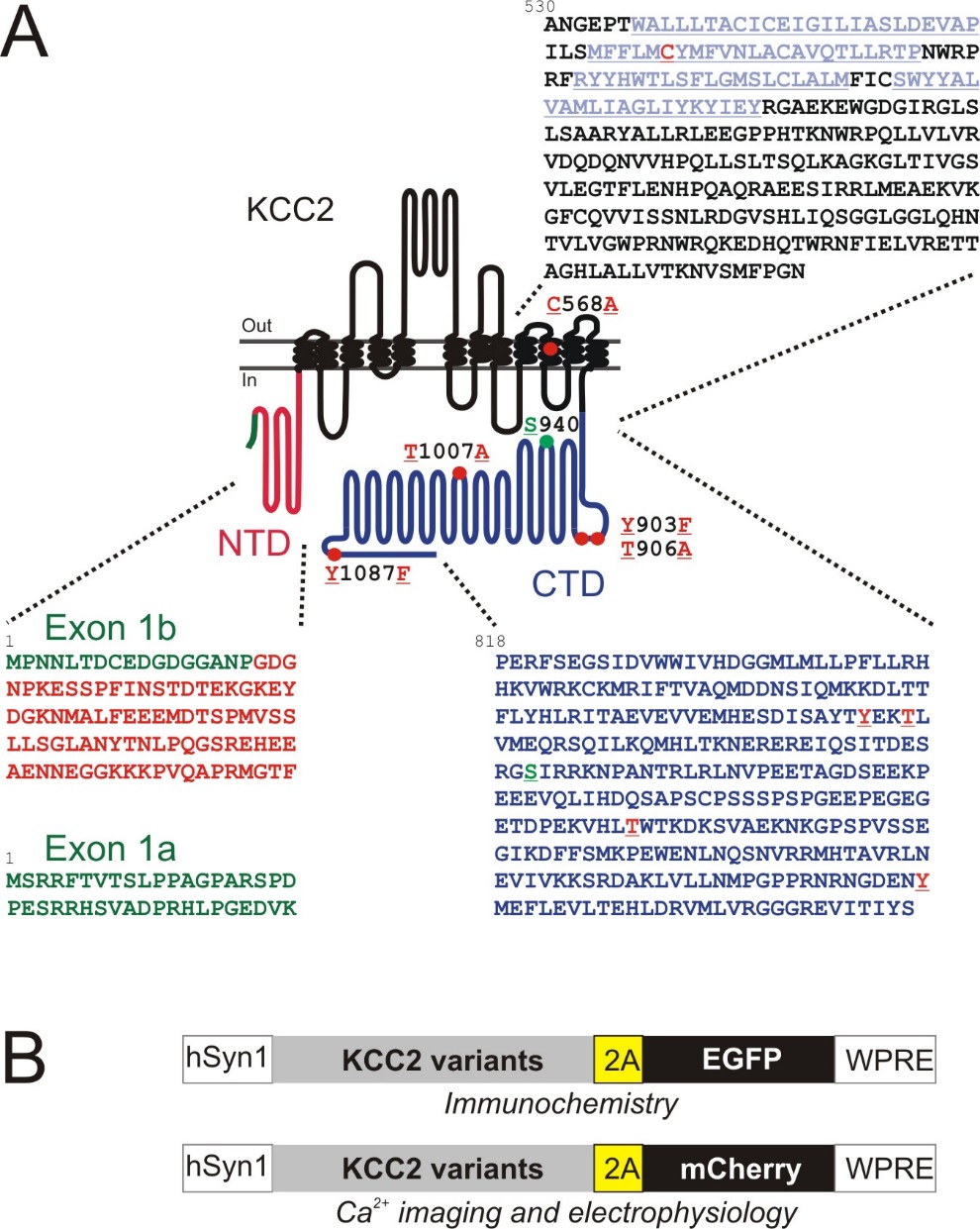


**SUPPL. FIGURE 1**


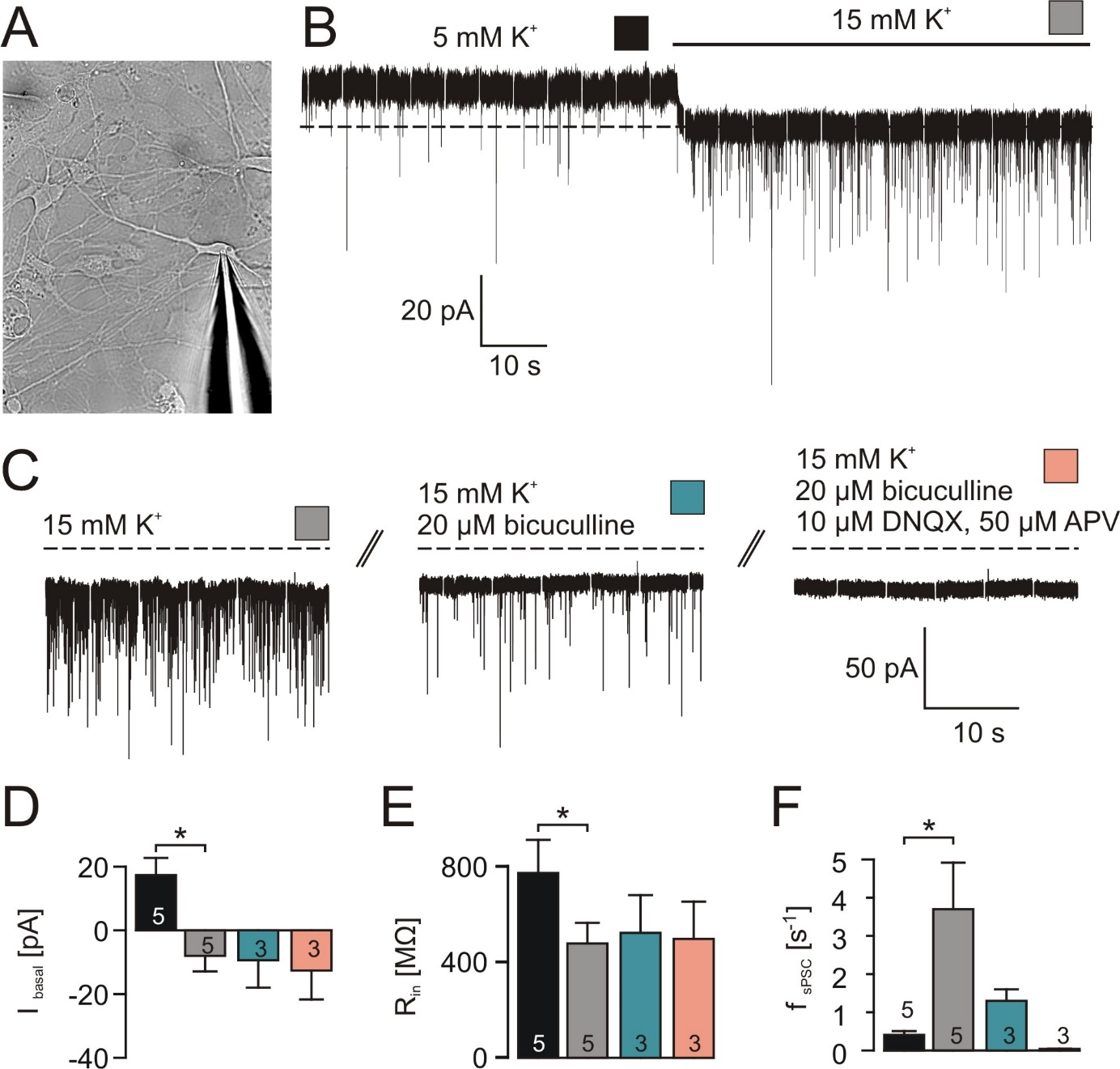


**SUPPL. FIGURE 2**

**
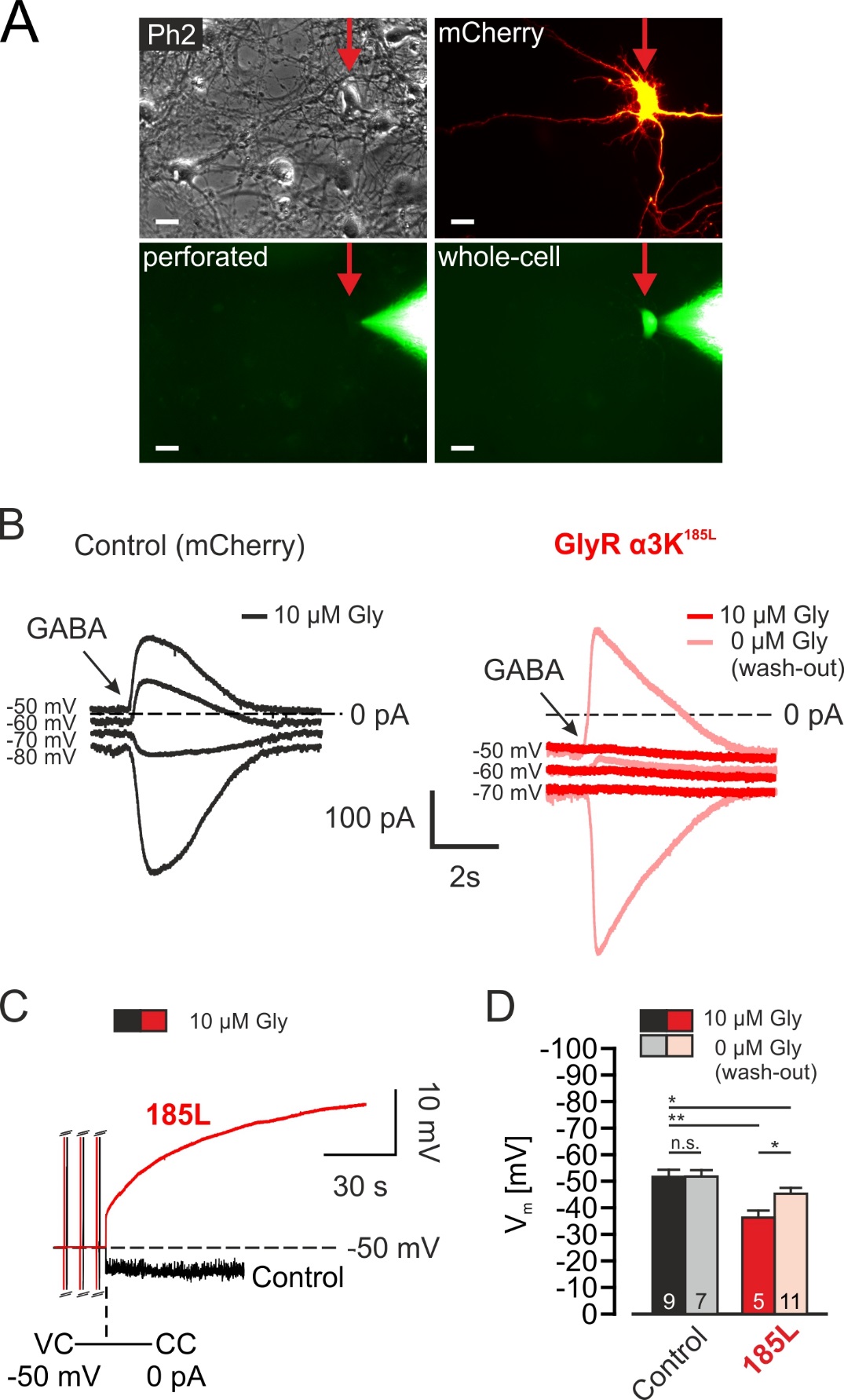
**

**SUPPL. FIGURE 3**


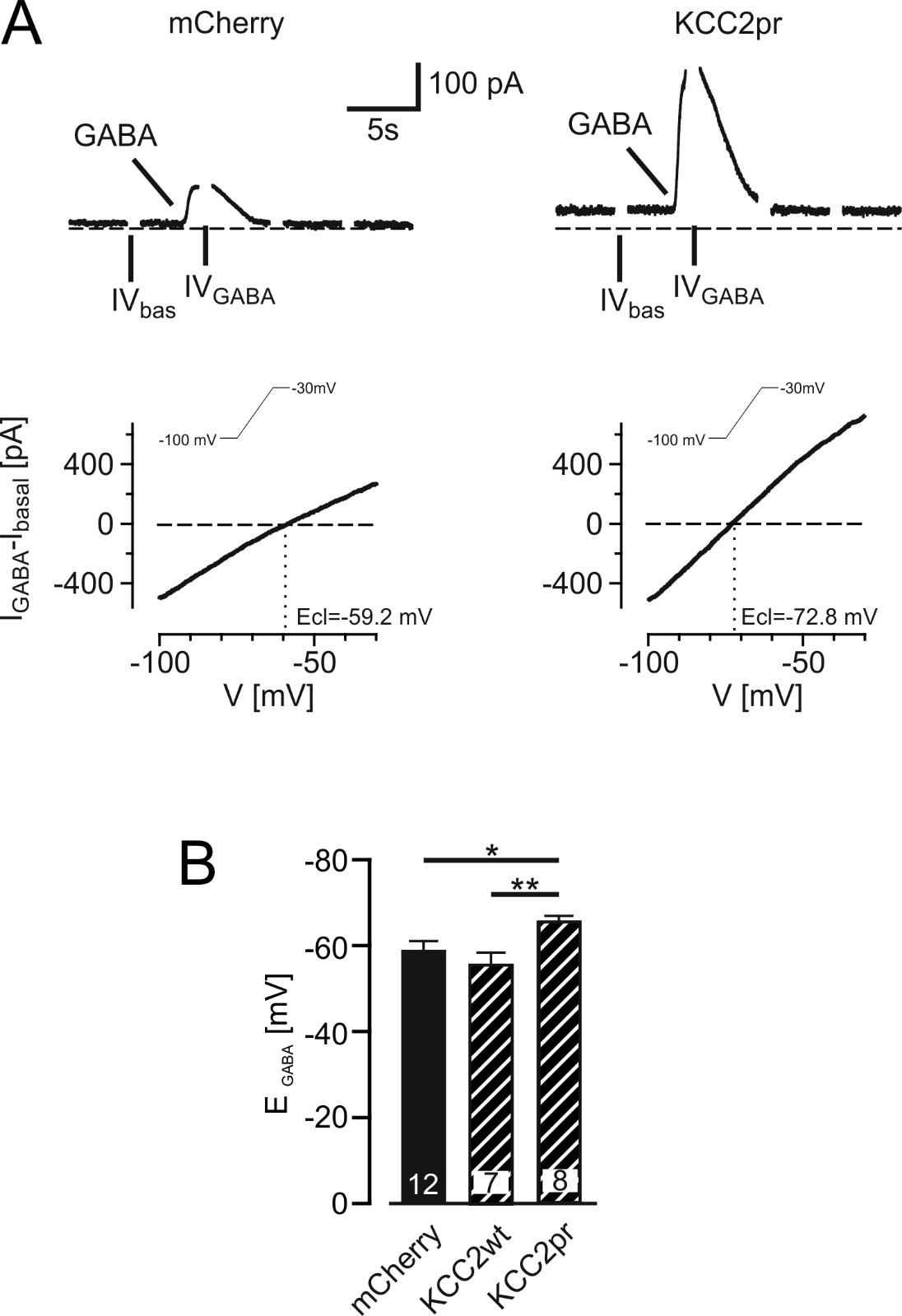


**SUPPL. FIGURE 4**


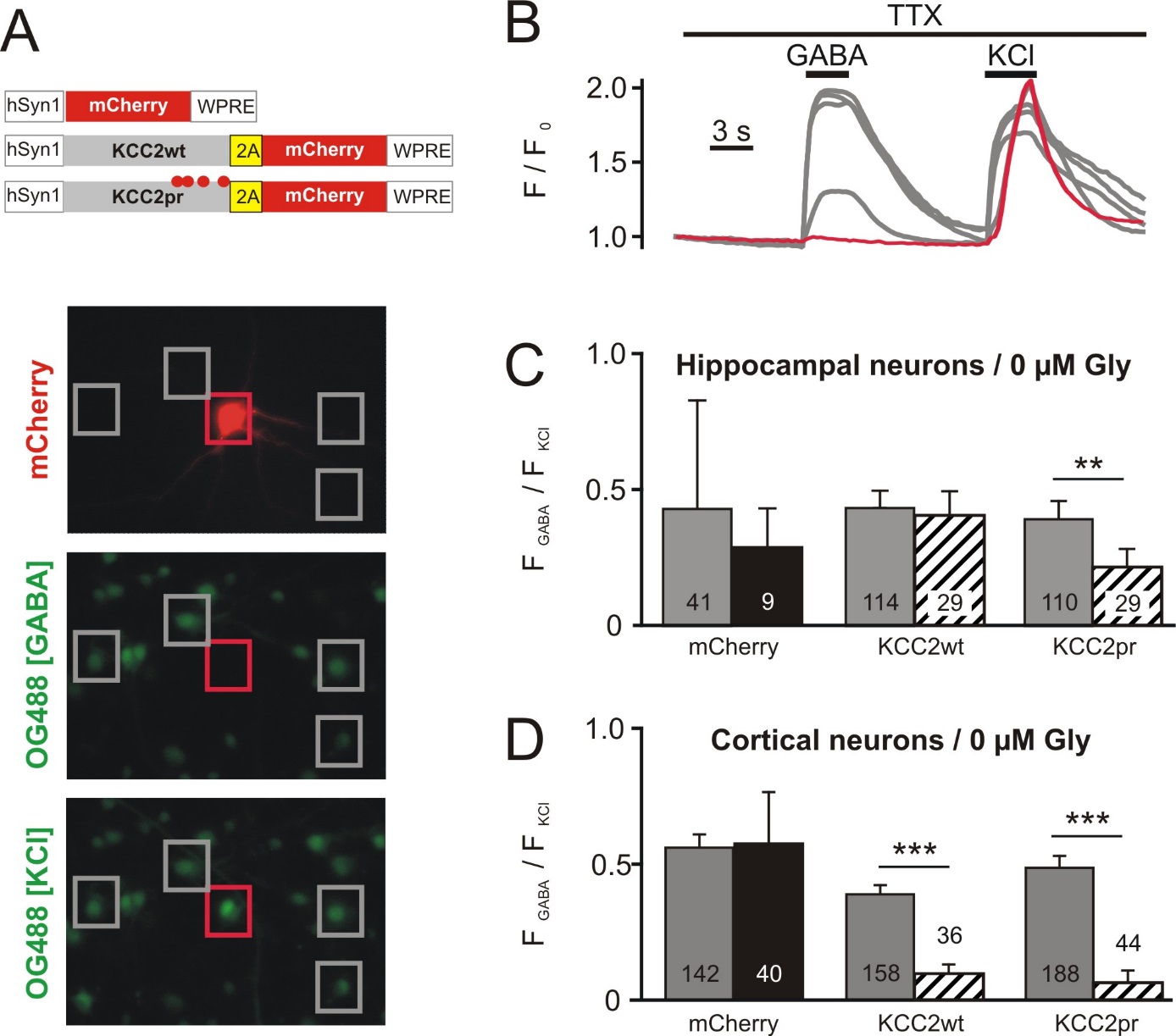


**SUPPL. FIGURE 5**


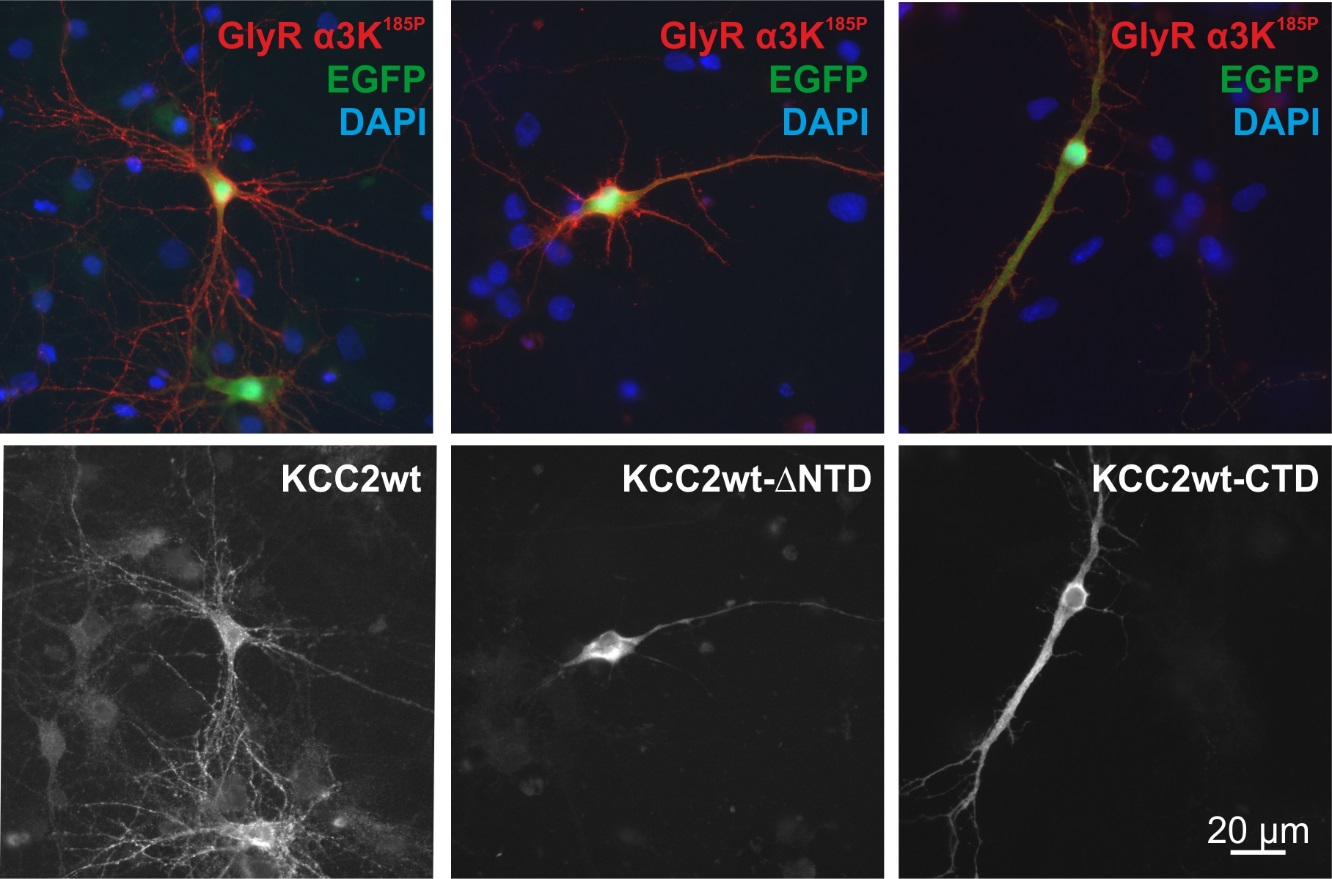


**SUPPL. FIGURE 6**
